# A Dedicated 21-Plex Proximity Extension Assay Panel for High-Sensitivity Protein Biomarker Detection Using Microdialysis in Severe Traumatic Brain Injury: The Next Step in Precision Medicine?

**DOI:** 10.1089/neur.2022.0067

**Published:** 2023-01-11

**Authors:** Philip Dyhrfort, Teodor Svedung Wettervik, Fredrik Clausen, Per Enblad, Lars Hillered, Anders Lewén

**Affiliations:** Department of Medical Sciences, Section of Neurosurgery, Uppsala University, Uppsala, Sweden.

**Keywords:** biomarker, cerebral microdialysis, neurointensive care, proximity extension assay, traumatic brain injury

## Abstract

Cerebral protein profiling in traumatic brain injury (TBI) is needed to better comprehend secondary injury pathways. Cerebral microdialysis (CMD), in combination with the proximity extension assay (PEA) technique, has great potential in this field. By using PEA, we have previously screened >500 proteins from CMD samples collected from TBI patients. In this study, we customized a PEA panel prototype of 21 selected candidate protein biomarkers, involved in inflammation (13), neuroplasticity/-repair (six), and axonal injury (two). The aim was to study their temporal dynamics and relation to age, structural injury, and clinical outcome. Ten patients with severe TBI and CMD monitoring, who were treated in the Neurointensive Care Unit, Uppsala University Hospital, Sweden, were included. Hourly CMD samples were collected for up to 7 days after trauma and analyzed with the 21-plex PEA panel. Seventeen of the 21 proteins from the CMD sample analyses showed significantly different mean levels between days. Early peaks (within 48 h) were noted with interleukin (IL)-1β, IL-6, IL-8, granulocyte colony-stimulating factor, transforming growth factor alpha, brevican, junctional adhesion molecule B, and neurocan. C-X-C motif chemokine ligand 10 peaked after 3 days. Late peaks (>5 days) were noted with interleukin-1 receptor antagonist (IL-1ra), monocyte chemoattractant protein (MCP)-2, MCP-3, urokinase-type plasminogen activator, Dickkopf-related protein 1, and DRAXIN. IL-8, neurofilament heavy chain, and TAU were biphasic. Age (above/below 22 years) interacted with the temporal dynamics of IL-6, IL-1ra, vascular endothelial growth factor, MCP-3, and TAU. There was no association between radiological injury (Marshall grade) or clinical outcome (Extended Glasgow Outcome Scale) with the protein expression pattern. The PEA method is a highly sensitive molecular tool for protein profiling from cerebral tissue in TBI. The novel TBI dedicated 21-plex panel showed marked regulation of proteins belonging to the inflammation, plasticity/repair, and axonal injury families. The method may enable important insights into complex injury processes on a molecular level that may be of value in future efforts to tailor pharmacological TBI trials to better address specific disease processes and optimize timing of treatments.

## Introduction

Traumatic brain injury (TBI) is a complex and heterogeneous condition with a high mortality and burden of long-term sequelae.^1,2^ Multi-modality monitoring, including intracranial pressure (ICP), cerebral perfusion pressure (CPP), brain tissue oxygenation, and brain energy metabolism (cerebral microdialysis; CMD), has been implemented in several neurointensive care (NIC) units to better understand the pathophysiological mechanisms and ultimately to give more refined treatments.^3–5^ In addition to derangements in these physiological variables, several other post-traumatic injury processes may occur. Particularly, the role of inflammation has received increased interest and understanding lately.^5–8^ Severe TBI may induce both systemic and central nervous system (CNS) inflammatory responses, and these two phenomena may interact, for example, by activation and migration of systemic immune cells into the CNS, facilitated by concurrent blood–brain barrier (BBB) damage and activation of parenchymal astrocytes and microglia.^7,9,10^ Neuroinflammation is considered a double-edged sword with both distinct beneficial, neuroprotective mechanisms, as well as severe detrimental, neurotoxic effects.^7^

There have been several attempts to influence inflammatory response by pharmacological agents, such as steroids, sex hormones, statins, and cell-cycle inhibitors, but with limited therapeutic success.^11^ There is now an increased interest in bedside monitoring of biomarker patterns, reflecting neuroinflammation, growth factors, and neuronal injury biomarkers, to better understand the underlying disease processes on a molecular level and, ultimately, be able to give more tailored pharmacological agents taking into account both timing and molecular mechanisms. By using modern CMD catheters with larger cut-off membranes, it is possible to continuously sample proteins from cerebral interstitial fluid.^12–16^ Together with the newly developed technique, the proximity extension assay (PEA),^6^ which is a very sensitive tool to detect small quantities of protein while using only small volumes of fluid, it is now also possible to efficaciously detect and study numerous brain protein biomarkers from patients with severe TBI.^17–19^

In a recent study by our group, we analyzed 92 potential protein biomarkers of inflammation from CMD samples of 10 patients with severe TBI, using PEA technology.^6^ In the present study, we customized a PEA panel prototype of 21 selected candidate proteins, involved in inflammation, neuroplasticity/-repair, and axonal injury in another cohort of 10 TBI patients. The aim was to explore their temporal dynamics and relation to age, type of brain injury, and clinical outcome.

## Methods

### Patient population and management

The Department of Neurosurgery at the University Hospital in Uppsala, Sweden, provides neurosurgical care for a central part of Sweden with a population of ∼2 million. Most patients are treated initially at local hospitals according to advanced trauma life-support principles and then transferred to Uppsala. This study included 10 patients with severe TBI, who were unconscious (Glasgow Coma Scale [GCS] Motor [GCS M] score <6), intubated and mechanically ventilated, and treated at our NIC unit with ICP and concurrent CMD monitoring of protein and metabolic biomarkers.

The management protocol has been described in detail in previous studies.^20,21^ Treatment goals were ICP ≤20 mm Hg, CPP ≥60 mm Hg, systolic blood pressure >100 mm Hg, pO_2_ >12 kPa, arterial glucose 5–10 mmol/L, electrolytes within normal ranges, normovolemia, and body temperature <38°C. Unconscious (GCS M, <6) patients were intubated, mechanically ventilated, and received an ICP monitor (intraparenchymal or an external ventricular drain [EVD]). Propofol was administered for sedation and morphine for analgesia. The head of the bed was elevated to 30 degrees. Intracranial lesions with significant mass effect were surgically evacuated. In situations of increased ICP, despite basal NIC treatments, and when no mass lesion was present, cerebrospinal fluid (CSF) was drained with an EVD. If ICP was still refractorily elevated, a thiopental infusion was started and, thereafter, a decompressive craniectomy (DC) was performed as a last resort.

### Clinical variables, radiological imaging, and outcome: data acquisition

Data on demographics, NIC admission variables, and treatments were extracted from the prospective Uppsala TBI register.^22^ The first computed tomography (CT) scan after injury was analyzed according to the Marshall classification,^23^ and a crude sorting of the most dominant cerebral injury visible on the first CT was also done for all patients. In cases where several types of injuries were equally present (e.g., traumatic subarachnoid hemorrhage, contusions, and subdural hematomas), the term “mixed” brain injury was used. Clinical outcome was evaluated 6 months post-injury, by specially trained personnel with structured telephone interviews, using the Extended Glasgow Outcome Scale (GOS-E), containing eight categories of global outcome, from death to upper good recovery.^24–26^

### Cerebral microdialysis monitoring

**FIG. 1.**
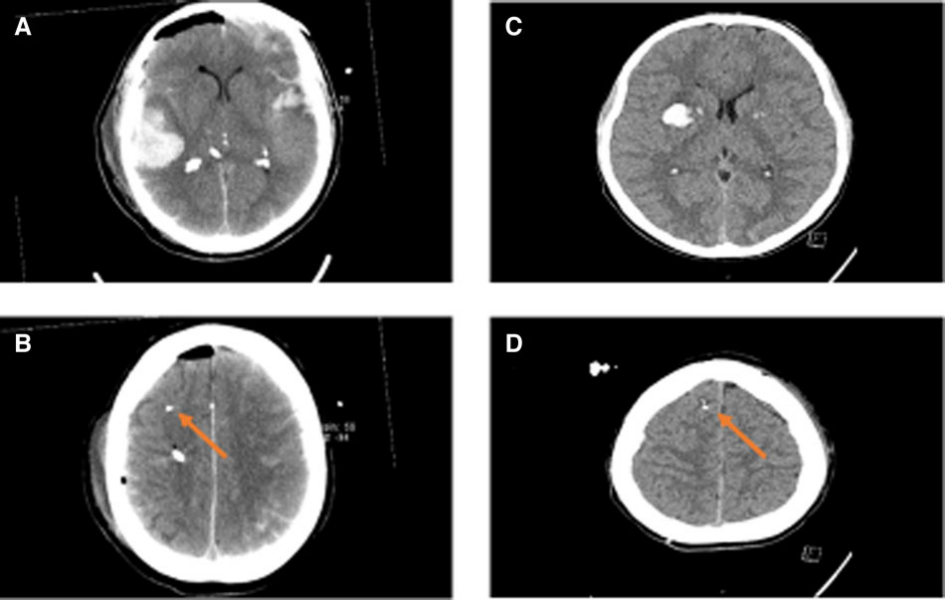
Computed tomography scans of case nos. 1 and 9—structural injuries and relation to the CMD catheter. The figure demonstrates the computed tomography scans of cases no. 1 (**A,B**) and no. 9 (**C,D**). Case no. 1 exhibited extensive mixed lesions of particularly cerebral contusions and traumatic subarachnoid hemorrhage (A). The cerebral microdialysis catheter (red arrow) was located in the injured lobe, but in the normal-appearing brain, distant from any hemorrhagic lesion (B). Case no. 9 exhibited a deep cerebral contusion (C). The CMD catheter (red arrow) was also located in the injured lobe, but in the normal-appearing brain, distant from any hemorrhagic lesion (D). CMD, cerebral microdialysis.

The CMD procedure has previously been described in detail in previous studies by our group.^6,27^ Briefly, the CMD catheter was inserted in conjunction with implantation of the ICP-monitoring device in the right frontal lobe (Fig. 1). The 71 High Cut-Off (100 kDa) CMD catheter was used with a membrane length of 10 mm (M Dialysis AB, Stockholm, Sweden). Artificial CSF was used as perfusion fluid, containing NaCl 147 mM, KCl 2.7 mM, CaCl_2_ 1.2 mM, and MgCl_2_ 0.85 mM, with the addition of 1.5% human serum albumin, at a perfusion rate of 0.3 μL/min delivered by a 106 Microdialysis pump (M Dialysis).

Sampling was started ∼1 h after insertion of the CMD catheter to allow for normalization of changes caused by catheter implantation. Cerebral metabolites (glucose, pyruvate, lactate, glycerol, and glutamate) were measured hourly and continuously evaluated during routine clinical care. Further samples (∼18 μL) for the multiplex PEA analysis were collected every third hour and stored at −70°C until protein biomarker analysis.

### Protein biomarker analysis

Levels of 21 potential protein biomarkers in CMD samples were measured every third hour in pooled consecutive, hourly CMD samples by the multi-plex PEA panel that was designed, constructed, and validated by our group in collaboration with Olink Proteomics (Olink, 21-Plex Custom Made Panel; Olink Proteomics AB, Uppsala, Sweden),^28^ based on the PEA results from our previously published TBI study.^6^ In brief, 1 μL of CMD sample was incubated with a set of paired antibodies where two oligonucleotide-conjugated antibodies would bind to the same protein. The affinity bindings of the antibodies brought the two attached oligonucleotides in proximity, allowing them to be extended using enzymatic DNA polymerization in the assay. The resulting double-strand DNAs were subsequently amplified and quantified by real-time quantitative polymerase chain reaction (qPCR) by a microfluidic PCR system (Fluidigm, San Francisco, CA).

Expression levels of the biomarkers were expressed as normalized protein expression (NPX). The NPX value is a relative unit of expression in log_2_ scale, resulting in that an increase of one unit of the value represents a doubling of the protein concentration in the sample. Values less than or equal to the limit of detection (LoD) were set to the estimated LoD and included in all analyses. For each protein, the optimal dilution was used. Draxin, transforming growth factor 1 (TGF1), granulocyte colony-stimulating factor (G-CSF), monocyte chemoattractant protein (MCP)-2, interleukin (IL)-1b, and urokinase-type plasminogen activator (uPA) were run at a 1:1 dilution and the others at 1:10. Characteristics of the selected proteins are described in Supplementary Table S1.

Precision of the 21-plex panel, analyzed as average Intra %CV (percent coefficient of variation) and Inter %CV based on two control CMD samples measured in triplicate on each of the 10 PEA plates used, was found to be 8% (Intra %CV) and 13% (Inter %CV), respectively.

### Statistical analysis

The study primarily 1) aimed to describe the temporal dynamics post-injury of this subset of candidate biomarkers in cerebral interstitial fluid and secondarily 2) to describe their relation to age, structural injury, and clinical outcome.

The protein profile was determined as the first 40 samples within the first 7 days post-injury. Each sample was pooled from consecutive 3-h windows, and 40 samples hence represented 120 h (only three missing samples in total). For example, if the first CMD sample was acquired on the 23rd hour post-injury, then hours 23–26 made up the first of the 40 samples for that patient. The temporal dynamics for each biomarker was illustrated with line plots and statistically described using cubic beta splines. Cross-correlation analyses were performed to assess the inter-relation among the studied proteins. Time-series analyses, taking into account time, age, and outcome, were done with linear mixed models. Exploratory principal component analysis (PCA) was conducted to explore eventual biomarker clusters depending on age, structural injury (Marshall grade), and outcome. *p* values from each test were adjusted for multiple testing using the Benjamin-Hochberg approach. An adjusted *p* value <0.05 was considered statistically significant.

### Ethics

The study was approved by the regional ethics board (2010/138 and 2010/138/1) and the Swedish Ethical Review Authority (2020-05462). Written informed consent was obtained either by the patients at follow-up or their closest relative during NIC.

## Results

### Demographics, admission variables, treatments, and clinical outcome

The patient cohort is described in detail in Table 1. Briefly, 10 patients (8 males and 2 females), with a mean age of 36 years (range, 11–71), with severe TBI were included. The injury panorama was equally divided between fall accidents and motor vehicle accidents (MVAs). Three of the patients were operated on because of epidural hematoma or contusions the first day post-injury. Three received an external ventricular drain on days 4–5 post-injury to lower ICP. Four patients developed a respiratory infection within the first 7 days.

**Table 1.** Demography, Admission Variables, NIC Variables, and Clinical Outcome

Case no.	1	2	3	4	5	6	7	8	9	10
Sex	Male	Male	Female	Male	Male	Female	Male	Male	Male	Male
Age (years)	71	56	45	51	12	15	21	11	13	65
Mechanism of injury	Fall	Fall	MVA	Fall	MVA	MVA	Fall	Fall	MVA	MVA
CT finding	Mixed	Mixed	Mixed	EDH	Contusion	Mixed	Mixed	Mixed	Contusion	tSAH
Marshall score (1–6)	6	3	2	5	3	5	5	3	3	2
Neurosurgical monitoring/intervention	ICP CMD	ICP CMD	ICP CMD	ICP CMD EDH evac.	ICP CMD	ICP CMD Contusion evac. DC	ICP CMD Contusion evac.	ICP CMD	ICP CMD	ICP CMD
MD-probe location	Normal brain	Normal brain	Normal brain	Normal brain	Normal brain	Contusion	Contusion	Normal brain	Normal brain	Normal brain
GCS M at admiss-ion	6	6	5	3	5	5	5	5	5	5
GCS M discharge	4	6	6	3	6	6	6	6	6	5
Length of stay in NIC (days)	12	18	35	12	17	18	24	14	12	18
MD start (h post-TBI)	7	35	28	21	7	11	14	9	8	10
GOS-E	Dead	Upper severe disability	Lower severe disability	Vegetative state	Upper severe disability	Lower severe disability	Lower severe disability	Good recovery	Upper severe disability	Lower moderate disability

The table includes characteristics of the 10 individual patients. Age span reached from 11 to 71 years. Mechanism of injury was either MVA or fall. GCS M was noted at admission and discharge. GOS-E was recorded at a follow-up ∼6 months after time of injury.

CMD = cerebral microdialysis; EDH = epidural hematoma; GCS M = Glasgow Coma Scale Motor score; GOS-E = Extended Glasgow Outcome Score; ICP = intracranial pressure; MVA = motor vehicle accident; NIC = neurointensive care; TBI = traumatic brain injury; tSAH = traumatic subarachnoid hemorrhage.

### Temporal dynamics and cross-correlations of the cerebral microdialysis biomarkers

**FIG. 2.**
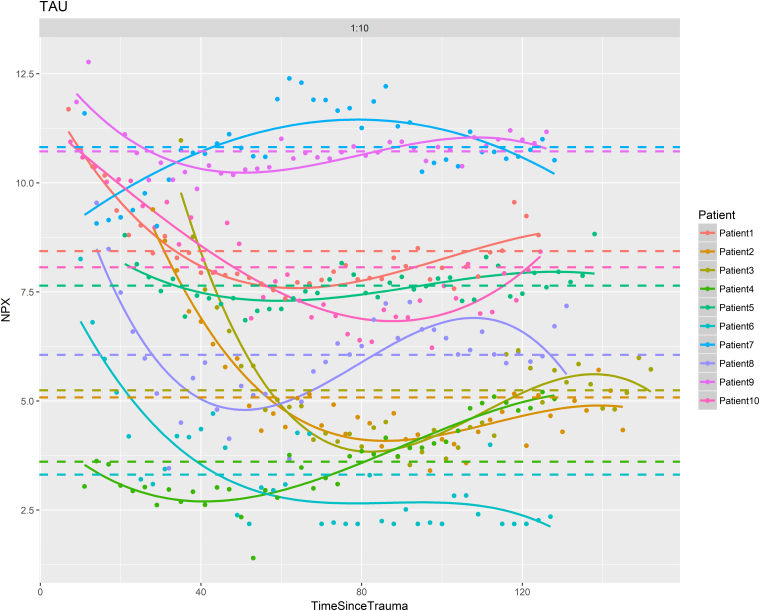
Temporal dynamics in TAU the first 7 days post-injury—a line-plot analysis. The figure demonstrates the temporal dynamics on TAU the first 7 days post-injury. There was a substantial difference in NPX between patients. The temporal trend also varied among patients, although NPX generally seemed to gradually decrease in most patients. NPX, normalized protein expression.

**FIG. 3.**
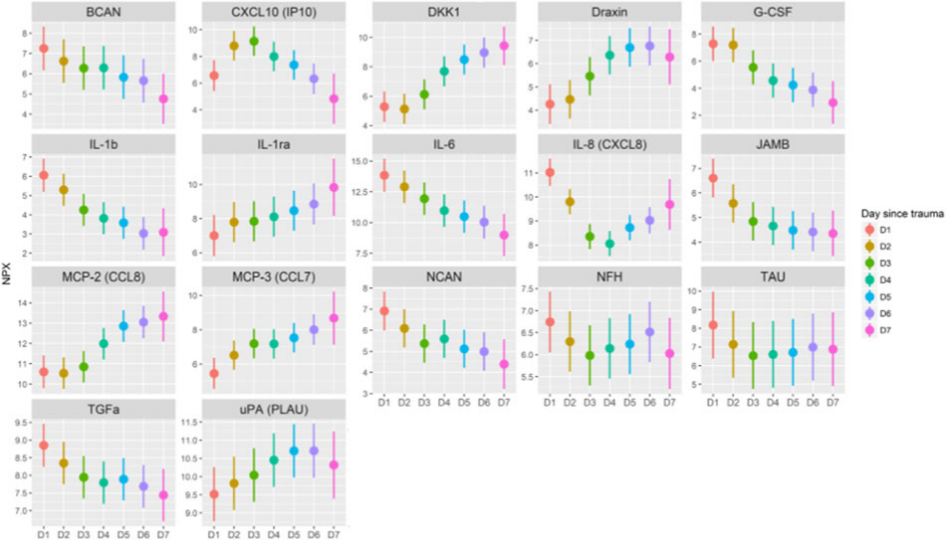
Temporal dynamics of protein biomarkers the first 7 days post-injury. The figure demonstrates those 17 of 21 proteins with significantly (adjusted *p* value, <0.05) different means between the days within the first 7 days post-injury. NPX values are on the y-axis and the day after injury on the x-axis. Dots indicate the mean value controlled for individual means. The line indicates the 95% confidence intervals as calculated by the model. NPX, normalized protein expression.

**FIG. 4.**
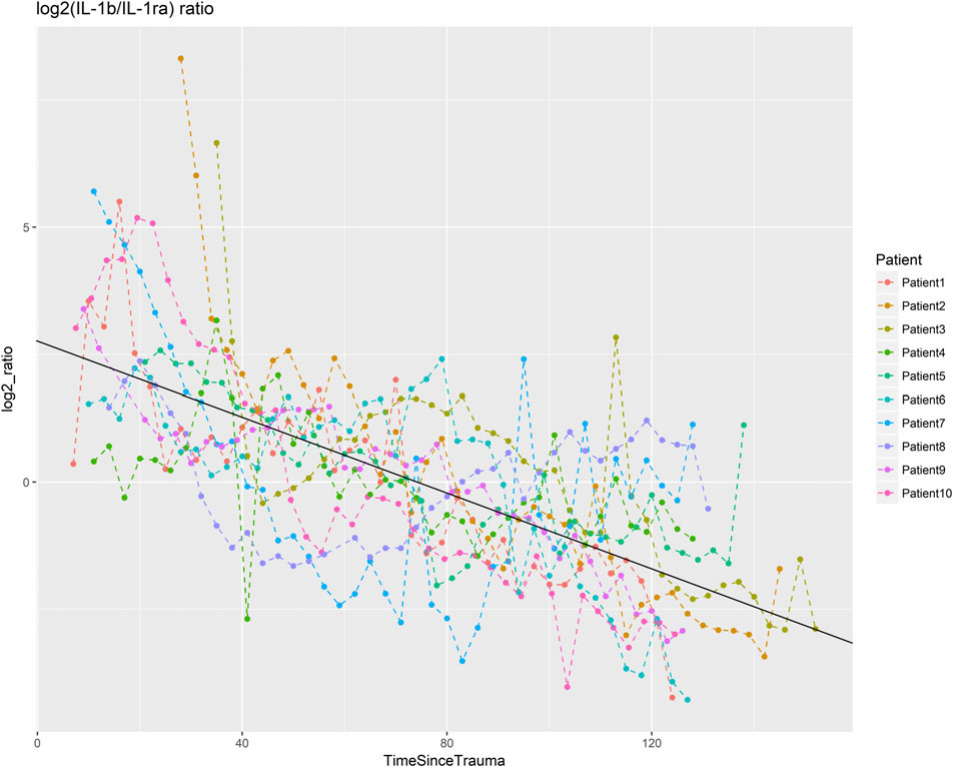
Temporal course of the log_2_ IL-1b/IL-1ra ratio. NPX values for IL1-b and IL1ra were centralized with the mean for each patient to better show the similarity of the decrease. The ratio between centralized IL1-β and IL-1ra was calculated and plotted with a log_2_ scale. A fixed-effect regression line is shown in black. The ratio shows a strong significant decrease in ratio post-injury (*p* < 10^–16^) of −0.037/h (log_2_ scale). IL, interleukin; IL-1ra, interleukin-1 receptor agonist; NPX, normalized protein expression.

The temporal dynamics of the CMD proteins was evaluated during the first 7 days post-injury. Figure 2 illustrates the temporal dynamics of TAU, and Supplementary Figures S1–S20 show the dynamics of the remaining 20 proteins in all 10 patients. Seventeen of the 21 proteins exhibited a significant temporal dynamic pattern (Fig. 3). Of these, brevican (BCAN), G-CSF, IL-1β, IL-6, junctional adhesion molecule B (JAM-B), neurocan (NCAN), and TGF-α showed early peaks (<48 h post-injury), C-X-C motif chemokine ligand 10 (CXCL10) mid-peaks (48–96 h post-injury), Dickkopf-related protein 1 (DKK1), Draxin, interleukin-1 receptor antagonist (IL-1ra), MCP-2, MCP-3, and uPA late peaks (96–168 hours post-injury), whereas neurofilament heavy chain (NFH), IL-8, and TAU showed biphasic peaks (Table 2). Only cluster of differentiation 200 (CD200), macrophage inflammatory protein (MIP)-1b, repulsive guidance molecule A (RGMA), and vascular endothelial growth factor (VEGF) exhibited stable values throughout the study period. In general, levels varied between patients, but the overall temporal trend for various biomarkers were strikingly similar (see Fig. 2 and Supplementary Figs. S1–S20). There was a significant decline in the log_2_ scaled ratio between IL1β and IL-1ra (Fig. 4). Cerebral energy metabolites during this period are demonstrated in Supplementary Table S2.

**Table 2.** Summary of Temporal Dynamics of the Individual Proteins

Early peak	Mid-peak	Late peak	Biphasic peak	Stable
BCAN	CXCL10	DKK1	NFH	CD200
G-CSF		Draxin	IL-8 (CXCL8)	MIP-1b (CCL4)
IL-1β		IL-1ra	TAU	RGMA
IL-6		MCP-2 (CCL8)		VEGF
JAM-B		MCP-3 (CCL7)		
NCAN		uPA		
TGF-α				

Early peak was defined as within 48h post-injury, mid-peak within the interval 48–96h post-injury, late peak as within the interval 96–150h post-injury, biphasic as peaks both in the early and late phase, and stable trends as no peaks.

BCAN, brevican; G-CSF, granulocyte colony-stimulating factor; IL, interleukin; JAM-B, junctional adhesion molecule B; NCAN, neurocan; TGF-α, transforming growth factor alpha; CXCL, C-X-C motif chemokine ligand; DKK1, Dickkopf-related protein 1; IL-1ra, interleukin-1 receptor agonist; MCP, monocyte chemoattractant protein; CCL, chemokine (C-C motif) ligand; uPA, urokinase-type plasminogen activator; NFH, neurofilament heavy chain; CD200, cluster of differentiation 200; MIP, macrophage inflammatory protein; RGMA, repulsive guidance molecule A; VEGF, vascular endothelial growth factor.

**FIG. 5.**
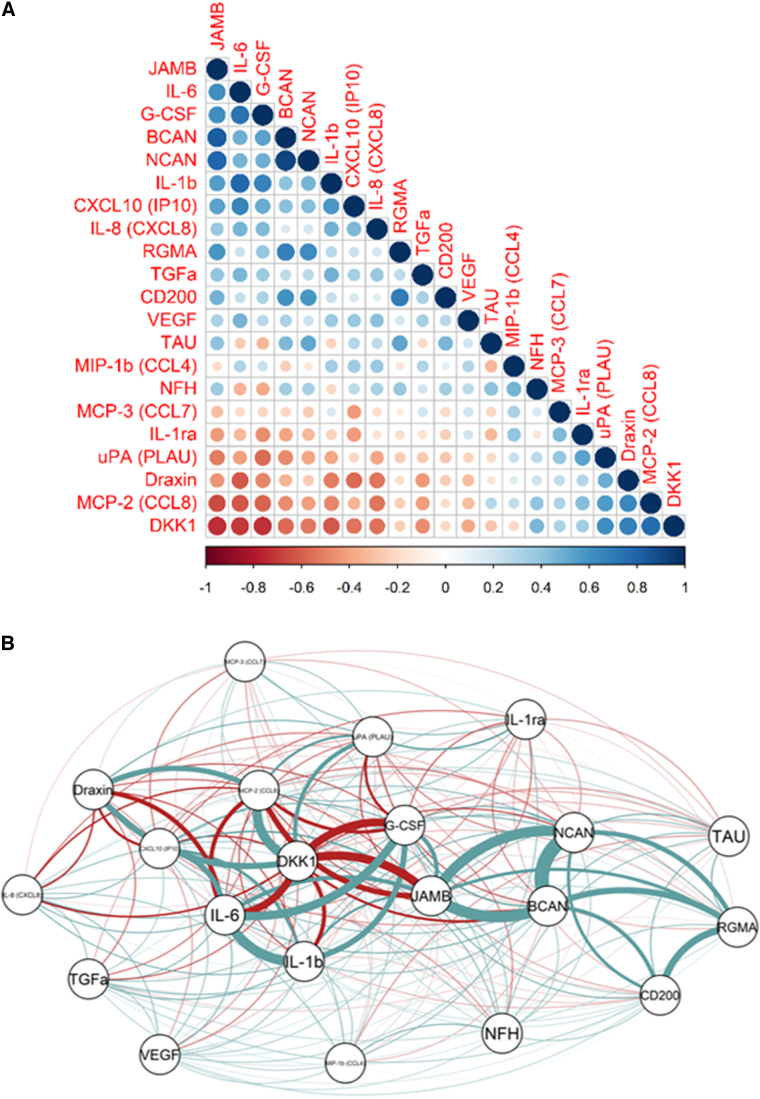
Cross-correlation among the cerebral microdialysis biomarkers. Both figures depict the relationships among the proteins, but in different ways. The correlation plot depicts the strength and direction of the associations among the proteins (**A**). The network depicts both the strength of correlation by thickness of the connecting line and similarity by proximity (**B**). For example, DKK-1 was inversely associated with IL-1β, IL-6, JAM-B, and G-CSF. DKK-1, Dickkopf-related protein 1; G-CSF, granulocyte colony-stimulating factor; IL, interleukin; JAM-B, junctional adhesion molecule B.

Cross-correlations among CMD proteins were assessed, as demonstrated in Figure 5. Both IL-1β and IL-6 were strongly associated with each other. JAM-B, BCAN, and NCAN also exhibited a strong association. DKK-1 was inversely associated with IL-1β, IL-6, JAM-B, and G-CSF.

### Cerebral microdialysis biomarkers in relation to age, type of brain injury, and clinical outcome

**FIG. 6.**
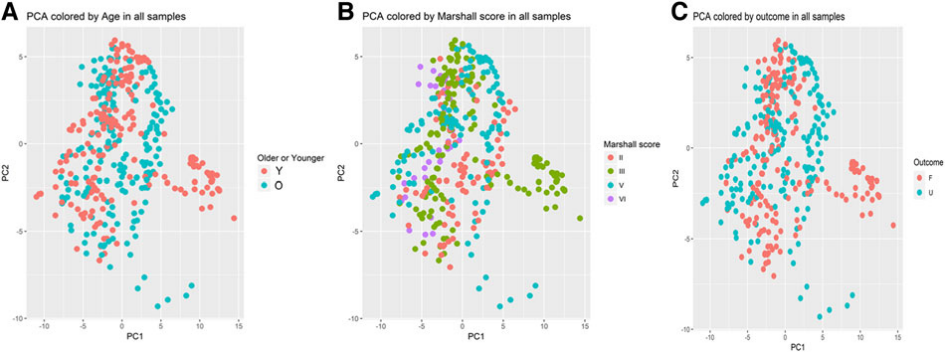
CMD biomarkers in relation to age, Marshall grade, and clinical outcome—an exploratory PCA analyses. The figures demonstrate exploratory PCA analyses for the CMD biomarkers in relation to old/young age above/below 22 years (**A**), Marshall grade (**B**), and favorable/unfavorable (GOS-E 5–8/1–4 [**C**]). PC1 and PC2 are the first and second principal directions with the greatest variation. CMD, cerebral microdialysis; GOS-E, Extended Glasgow Outcome Scale; PCA, principal component analysis.

**FIG. 7.**
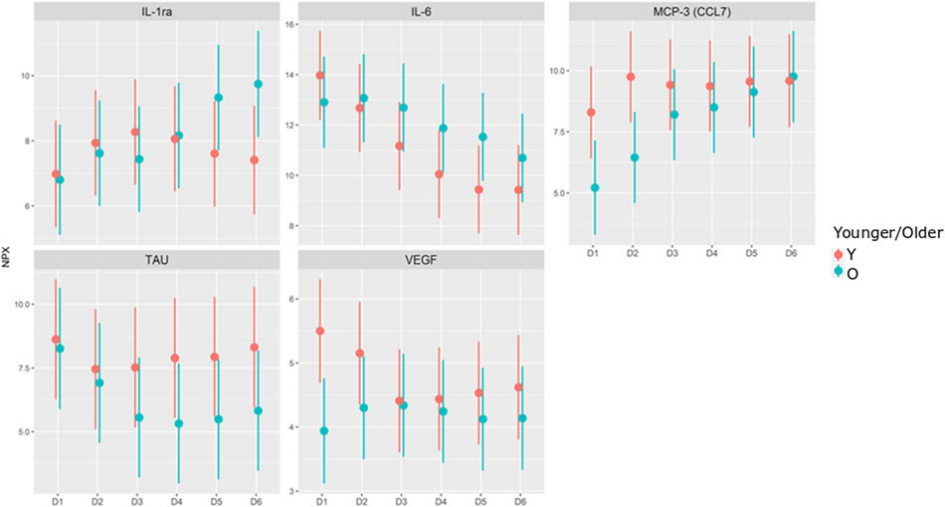
Time-series analyses: time-age interaction. Age showed a significant (adjusted *p* value, <0.05) interaction in a time series in these 5 of 21 biomarker proteins from the CMD samples. NPX values are shown on the y-axis, and the day after injury is shown on the x-axis. CMD, cerebral microdialysis; NPX, normalized protein expression.

In PCA analyses, there was no specific CMD protein pattern in relation to age, structural injury (Marshall grade), or clinical outcome (Fig. 6). In time-series analyses, there was an age interaction, so that younger patients tended to exhibit lower IL-1ra and IL-6 in the later course, but higher MCP-3 and VEGF in the early course and higher TAU in the late course (Fig. 7). In similar time-series analyses, there was no interaction of clinical outcome for any of the CMD proteins (data not shown).

## Discussion

A second generation of membranes for CMD catheters with larger pores have made it possible to reliably capture proteins from inside the BBB. These proteins can be measured using multi-plex assays of the CMD samples so that these potential biomarkers can be studied in great detail. In this study, the usefulness of a novel 21-plex protein assay for candidate protein biomarkers involved in neuroinflammation and cell survival in CMD samples from patients with severe TBI was studied. The main findings are a description of interesting protein dynamics that are hypothesis-generating regarding different aspects of the injury development and recovery in the acute phase after TBI. There was a striking similarity between individual patients regarding overall temporal protein level profiles, indicating that the results could be generalized to other patients with TBI. We believe that detailed protein profiling of cerebral CMD samples will increase our knowledge about important secondary injury mechanisms, which may be crucial for development of novel therapeutics and better disease characterization.

### Cell adhesion molecules

Cell adhesion molecules are of importance in the CNS regarding neuronal migration, axon-bundle formation, synapse formation, and formation of glial networks. The complex network of adhesion lays the structure for brain morphology as well as coordination of brain functions.^29,30^

Two cell adhesion molecules were investigated in this study. First, the well-known protein, TAU, was one of the proteins in our panel. TAU is known to be involved in microtubule assembly and stabilization of neurons and axons.^31^ TAU has been proposed as a potential biomarker for brain injury in CSF^32^ and CMD samples.^33,34^ Consistent with previous studies,^34^ we found a temporal pattern characterized by an early peak in most patients and almost all dropped in mid-phase, with a slight increase during the last 2 days of measurement. This early increase likely reflected the immediate primary brain injury. Increased TAU has previously been associated with focal lesions,^33^ but we did not find any association with TAU and Marshall grade. Magnoni and colleagues^34^ have previously found a significant association between higher CMD-TAU and worse outcome, but this was not observed in our study, possibly attributable to patient heterogeneity and a slightly smaller patient cohort in our material (*n* = 10 vs. 16). Second, we also studied JAM-B. JAM-B is an endothelium-specific adhesion molecule predominantly localized in the CNS. It has been associated with certain BBB functions, such as infiltration of cells into the parenchyma,^35^ and may reflect a compromised BBB.^36^ In this study, TBI patients exhibited early JAM-B elevations, which steadily decreased during the first 3 days in almost all patients, possibly reflecting gradual BBB recovery.

### Cytokines and chemokines

Cytokines are a large family of small proteins involved in cell signaling, which consist of interleukins, interferons, chemokines, lymphokines, tumor necrosis factors, and the colony-stimulating factors (hematopoietic growth factors).

Hematopoietic growth factors may exert neuroprotective effects, such as neural tissue repair and neurovasculogenesis.^37^ G-CSF is one of these factors, which is known to be involved in neuronal and endovascular regeneration after injury.^38^ Here, we observed that G-CSF was immediately elevated and gradually decreased. The early increase in G-CSF could be a response to the initial injury, followed by a normalization. This temporal pattern was also consistent with earlier studies by Helmy and colleagues.^16,39^

IL-1β is a proinflammatory cytokine, which seems to exert a negative effect on the brain after TBI.^40^ Consistent with both experimental^41,42^ and clinical studies,^16,43,44^ IL-1β peaked early and gradually decreased in our material. IL-1ra is the endogenous antagonist that exerts opposite effects compared to IL-1α and IL-1β. IL-1ra is anti-inflammatory, and several studies suggest that it may ameliorate neuroinflammation^45,46^ and benefit neurological recovery^40^ after TBI. In this study, IL-1β and IL-1ra showed inverse trends over the temporal course, with a steadily decreasing ratio between the two subsequent to each day post-injury, indicating a gradual shift toward anti-inflammation. Despite the findings in previous studies regarding IL-1β and IL-1ra with worse/better clinical outcome, this was not replicated in this study. However, we advocate further exploration of the usefulness of the IL-1β/IL-1ra ratio as an inflammatory index in NIC.

IL-6 mostly exerts a proinflammatory effect after TBI. Consistent with previous clinical reports,^14,16,39,44,47^ IL-6 peaked early and gradually declined in most patients in the current study. IL-6 is known to be stimulated by IL-1β and, in line with this, exhibited a similar temporal trend given that IL-1β and the cross-correlation analysis also demonstrated a strong positive association between these two interleukins. The association between IL-6 in CNS after TBI and outcome are inconsistent. One CMD study found that elevated IL-6 correlates with more favorable outcome,^48^ but CSF studies generally indicate the opposite association,^5^ whereas no association with outcome was found in our data with CMD samples.

IL-8, also called CXCL8, is a chemokine that attracts primarily neutrophils, but also other granulocytes. High CMD levels have been observed in our previous TBI study, as well as in a study by Helmy and coworkers.^6,16^ The current results confirm the earlier findings, and we found a temporal biphasic pattern. We interpret the early peak to indicate signaling for chemotaxis and the later peak as connected to the phagocytosis of cellular debris.^6^

CXCL10 is a chemokine that is produced in many cell types as a response to interferon-γ and is responsible for macrophage or microglia recruitment into injured tissue.^49^ We found a peak around day 3 post-injury in the current study, which is in line with previous CMD studies.^6,16^ This peak likely indicates the time point when the brain parenchyma is under stress and signal to the target cells to migrate to the injured area.^50^ As a potential target for anti-inflammatory intervention in TBI, the delayed peak of CXCL10 appears attractive in comparison to, for example, IL-1β.^6^

Members of the MCP family form a major component of the CC family of chemokines and are considered the principal chemokines involved in the recruitment of monocytes/macrophages and activated lymphocytes.^51^ Especially, MCP-2 (chemokine [C-C motif] ligand [CCL] 7), but also MCP-3 (CCL8), showed a steady increase over time. These two chemokines have not been widely studied in the context of brain injuries, but in an experimental model of endotoxin-induced encephalitis, the dynamic pattern was the opposite, with an early peak and rapid decrease in concentration.^52^ Presently, the increase over time could be a sign that the tissue continues to be under stress and is signaling to the immune system for help to resolve the situation.

MIP-1b (CCL4) is produced by macrophages and microglia in response to tumor necrosis factor alpha and IL-1β stimulation and may, among other things, act as a chemotactic factor. We observed a relatively stable trend during the monitoring period. In a mouse model of focal TBI, Ciechanowska and colleagues found a 75-fold increase in the messenger RNA expression of MIP-1b 24 h after TBI in mice compared to sham controls, which translated into a 2-fold increase in protein concentration at 24 h.^53^ The increase in protein concentration remained high in the injured cortex for at least 7 days after injury in their study. Together, these data suggest that MIP-1B is rapidly increased and stays elevated for both the acute and subacute phase of the injury process.

### Growth factors and neurotrophins

TGF-α is a member of the epidermal growth factor family and is involved in proliferation, differentiation, and development. TGF-α exhibits neurotropic properties that protect neurons from various neurotoxic insults.^54^ In this study, TGF-α peaked early and showed a steady decrease, which could reflect an immediate protective response to the primary brain injury.

VEGF is primarily involved in the development and formation of blood vessels. It also acts as a chemotactic factor for macrophages and activates resting astrocytes.^8^ Previous studies by our group^6^ and Mellergård and colleagues^14^ have shown VEGF elevations after TBI, in which Mellergård and colleagues found that patients >25 years of age exhibited higher values.^15^ In the present study, we report generally lower and no temporal dynamics in VEGF levels as compared to our earlier study,^6^ in which some patients showed distinct peaks. In addition, in contrast to Mellergård and colleagues,^15^ older rather than younger patients exhibited lower VEGF levels. Heterogeneity in primary and secondary injury patterns and small patient cohorts could perhaps explain some of the conflicting results.

BCAN is a proteoglycan found on neurons, which is important during the development of the CNS, and is involved in the communication between neurons and the extracellular matrix (ECM) of the adult brain.^55^ Minta and colleagues have studied fragments of BCAN after TBI in CSF samples collected from EVD and found consistently higher levels in patients with unfavorable outcome.^56^ Here, we found a gradual decrease over time, suggesting an early peak after the acute injury followed by a slow recovery. NCAN is, similarly to BCAN, a proteoglycan found in the ECM of the CNS and is involved in the development of the brain.^57^ Minta and colleagues measured NCAN in the CSF from EVD from TBI patients, but found no relation to clinical outcome.^56^ Our data show a consistent decline over time, very similar to the dynamic of BCAN. One interpretation could be that, as the brain recovers, less NCAN is shed from the ECM.

Draxin is a repulsive axonal guidance protein that is involved in the development of many CNS structures.^58^ Currently, the importance of draxin in the adult brain is not yet elucidated. The protein is clearly important during development, but knocking draxin out in mice is not lethal, although animals exhibit axonal aberrations.^59^ In the present study, draxin gradually increased and plateaued after 5 days post-injury. One explanation could be that the need for proteins that repel axonal growth, as the brain starts to recover, gradually increases, in order to minimize the risk for faulty connections.

RGMA is another repulsive axonal guidance protein that is important during development. In a recent publication, Liu and colleagues showed that a decrease in methylation of RGMA in the CSF samples from TBI patients correlated with intracranial hypertension.^60^ Our data showed fairly stable levels during the time studied, and it is possible that the methylation of RGMA is a better biomarker than the levels of the protein itself.

### Other biomarkers

CD200 is expressed in the membrane of neurons and is an important “off-signal” to immune activation.^61^ Our data show low and stable levels over time, suggesting either no regulation after injury or that the protein does not enter the interstitial space.

uPA is a serine protease that converts inactive plasminogen to active plasmin, which then degrades fibrin clots. It has been proposed as a biomarker in TBI, but it is ubiquitous throughout the body and may also be released after, for example, extracranial injuries.^62^ Our results show an increase from day 1 to day 6 and then a decrease to day 7. This could reflect a resolving of microembolism in the injured brain requiring plasmin activity initiated by the release of uPA.

DKK1 is a cysteine-rich protein involved in normal embryonic development, but is also believed to be a marker of ongoing inflammation. DKK1 serum concentration has been reported to correlate with TBI severity and 30-day mortality.^63^ Here, we report a suggested biphasic dynamic of the protein with an early small peak at day 1, followed by a small dip in level on day 2, and then a distinct increase from day 3 to day 6 with a tapering off of the increase to day 7. This could be a sign of an increase over time for a certain aspect of the immune response to the injury.

NFH is an intermediate filament protein that makes up microfilaments and -tubules forming the neuronal cytoskeleton. The family of neurofilaments has been identified as promising biomarkers for several degenerative conditions to the brain given that an increase in CSF or blood indicates a degradation of neurons.^64^ Our data demonstrated a clear biphasic dynamic with an initial decrease from day 1 to day 3, followed by an increase that peaked on day 6. The initial decrease could reflect an early release of degraded cytoskeletons from necrotic neurons that resolves during the first days after injury. The later increase could reflect delayed apoptosis and/or greater production of the protein in the repair process.

### Methodological considerations

The strength of this study is the description of a novel combination of techniques (large-pore CMD with PEA) for analysis of focal cerebral biochemistry to study the complex interplay of injury and recovery processes that occur on a molecule level. This method carries a great advantage in combining multiple samples of the interstitial cerebral fluid from each patient and requires only a small fluid volume. This is beneficial, given that multiple samples increased the reliability as well as the sensitivity to detect temporal dynamic changes of the biomarkers in these patients. However, some limitations of the study include the small number of patients, heterogeneity in demographics and primary injuries, differences in clinical course during NIC, medications, and the focal nature of the CMD measurements with potentially limited global validity. Still, this field of study on multiple protein biomarkers is still growing, and our patient cohort was comparable to the limited numbers in previous studies.^5^ Last, the relation between systemic and neuroinflammation is important; however, although we had plasma biomarkers in some patients, we abstained from further analyses because of too small a sample size.

## Conclusion

There is a need to better understand the injury and recovery processes on a molecular level after severe TBI. The high cut-off CMD membrane with the PEA technique for sample analyses enabled us to use small volumes of cerebral interstitial fluid in order to study a large set of important protein biomarkers with high temporal resolution as well as sensitivity and specificity. In this pilot study using a newly designed 21-plex PEA panel, several biomarkers known to be involved in, for example, neuronal/axonal injury and inflammation exhibited significant, temporally dynamic changes the first 7 days post-injury. There was no clear association between biomarker pattern and the degree of structural injury or clinical outcome, but age tended to interact with some of the biomarkers. The study was limited by the small patient cohort. In future efforts, we intend to proceed with data collection and include more patients to attain more reliable results and be able to more thoroughly explore the factors responsible for certain biomarker patterns and their relation to clinical course and patient outcome.

## Supplementary Material

Supplemental data

Supplemental data

Supplemental data

## Abbreviations Used

BBB: blood–brain barrier
BCAN: brevican
CCL: chemokine (C-C motif) ligand
CD200: cluster of differentiation 200
CMD: cerebral microdialysis
CNS: central nervous system
CPP: cerebral perfusion pressure
CSF: cerebrospinal fluid
CXCL: C-X-C motif chemokine ligand
DC: decompressive craniectomy
DKK1: Dickkopf-related protein 1
ECM: extracellular matrix
ICP: intracranial pressure
IL1-ra: interleukin-1 receptor antagonist
JAM-B: junctional adhesion molecule B
LoD: limit of detection
MCP: monocyte chemoattractant protein
MIP: macrophage inflammatory protein
MVA: motor vehicle accident
NCAN: neurocan
NFH: neurofilament heavy chain
NIC: neurointensive care
NPX: normalized protein expression
PCA: principal component analysis
PEA: proximity extension assay
%CV: percent coefficient of variation
qPCR: quantitative polymerase chain reaction
RGMA: repulsive guidance molecule A
TBI: traumatic brain injury
TGF: transforming growth factor
uPA: urokinase-type plasminogen activator
VEGF: vascular endothelial growth factor
