# Supplementary material for: A Dedicated 21-Plex Proximity Extension Assay Panel for High-Sensitivity Protein Biomarker Detection Using Microdialysis in Severe Traumatic Brain Injury: The Next Step in Precision Medicine?
Source: Neurotrauma Rep. 2023 Jan 11;4(1):25–40. doi: 10.1089/neur.2022.0067 (PMC9886191; doi:10.1089/neur.2022.0067)
Supplement: Supplemental data [file Suppl_TableS1.docx]

**Supplementary Table 1. List of the 21 biomarkers**

| **Full name (shortening)** | **Classification** |
| --- | --- |
| Brevican core protein (BCAN) | neurotrophic factor |
| OX-2 membrane glycoprotein (CD200) | immunoglobin superfamily |
| C-X-C motif chemokine 10 (CXCL10 (IP10)) | chemokine |
| Dickkopf-related protein 1 (DKK1) | inhibitor of Wnt/β-catenin signaling |
| DRAXIN (Draxin) | neurotrophic factor |
| Granulocyte colony-stimulating factor (G-CSF) | cytokine |
| Interleukin-1 beta (IL-1b) | cytokine |
| Interleukin-1 receptor antagonist protein (IL-1ra) | interleukin superfamily |
| Interleukin-6 (IL-6) | interleukin superfamily |
| Interleukin-8 (IL-8 (CXCL8)) | chemokine |
| JAM2 blocking peptide (JAMB) | cell adhesion molecule |
| C-C motif chemokine 8/Monocyte chemotactic protein 2 (MCP-2 (CCL8)) | chemokine |
| C-C motif chemokine 7/Monocyte chemotactic protein 3 (MCP-3 (CCL7)) | chemokine |
| C-C motif chemokine 4 (MIP-1b (CCL4)) | chemokine |
| Neurocan core protein (NCAN) | neurotrophic factor |
| Neurofilament heavy polypeptide (NFH) | neurofilament |
| Repulsive guidance molecule A (RGMA) | neurotrophic factor |
| Microtubule-associated protein tau (TAU) | cell adhesion molecule |
| Transforming growth factor alpha (TGFa) | growth factor |
| Urokinase-type plasminogen activator (uPA  (PLAU)) | serine protease |
| Vascular endothelial growth factor A (VEGF-A) | growth factor |

List of proteins included in the 21-plex PEA panel used in this study.
