# Supplementary material for: A Dedicated 21-Plex Proximity Extension Assay Panel for High-Sensitivity Protein Biomarker Detection Using Microdialysis in Severe Traumatic Brain Injury: The Next Step in Precision Medicine?
Source: Neurotrauma Rep. 2023 Jan 11;4(1):25–40. doi: 10.1089/neur.2022.0067 (PMC9886191; doi:10.1089/neur.2022.0067)
Supplement: Supplemental data [file Suppl_FigS1-S20.docx]

**Supplementary Figure 1. Temporal dynamics in BCAN the first 7 days post-injury – a line plot analysis**


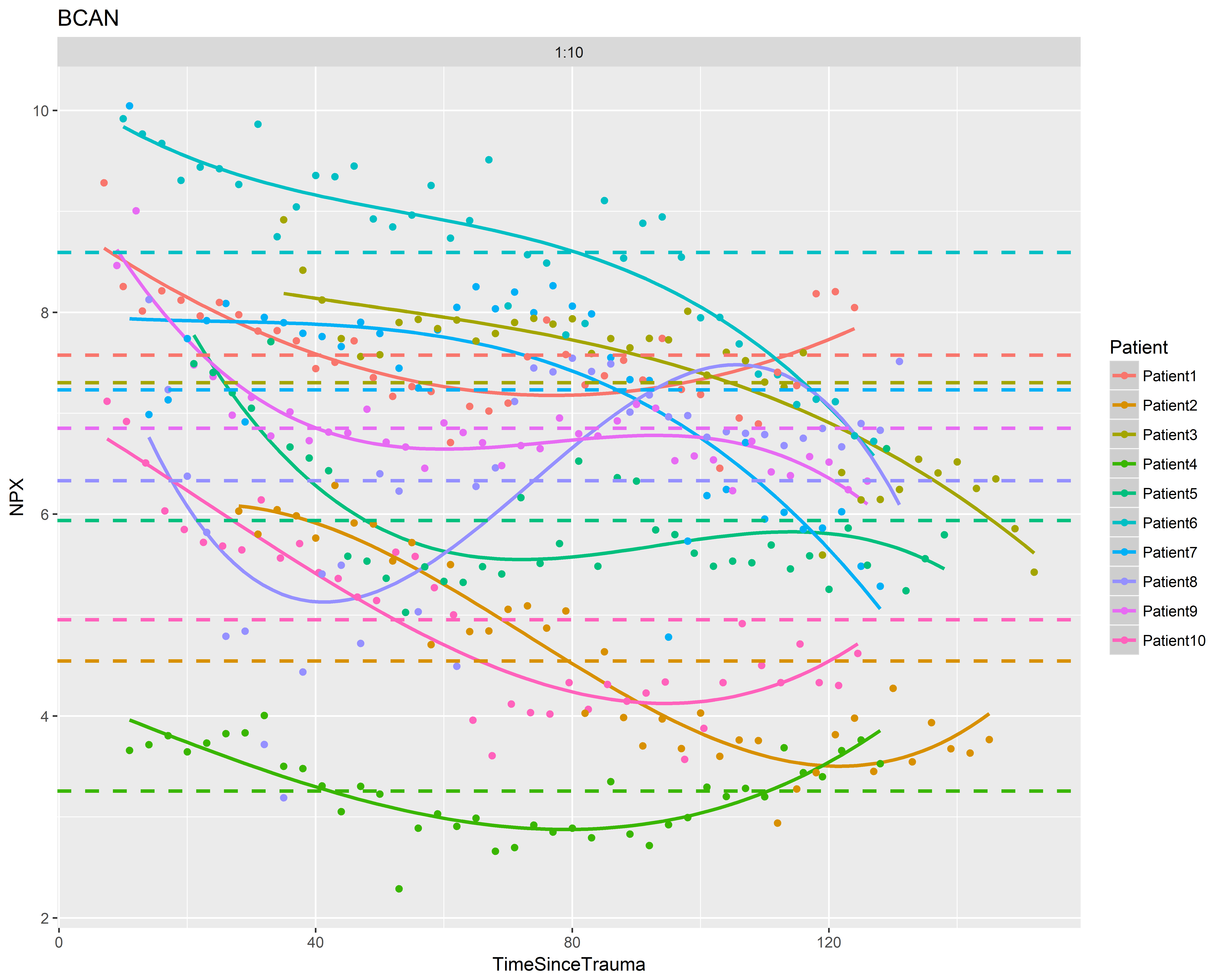


The figure demonstrates the temporal dynamics of BCAN the first 7 days post-injury in all 10 patients.

**Supplementary Figure 2. Temporal dynamics in CD200 the first 7 days post-injury – a line plot analysis**


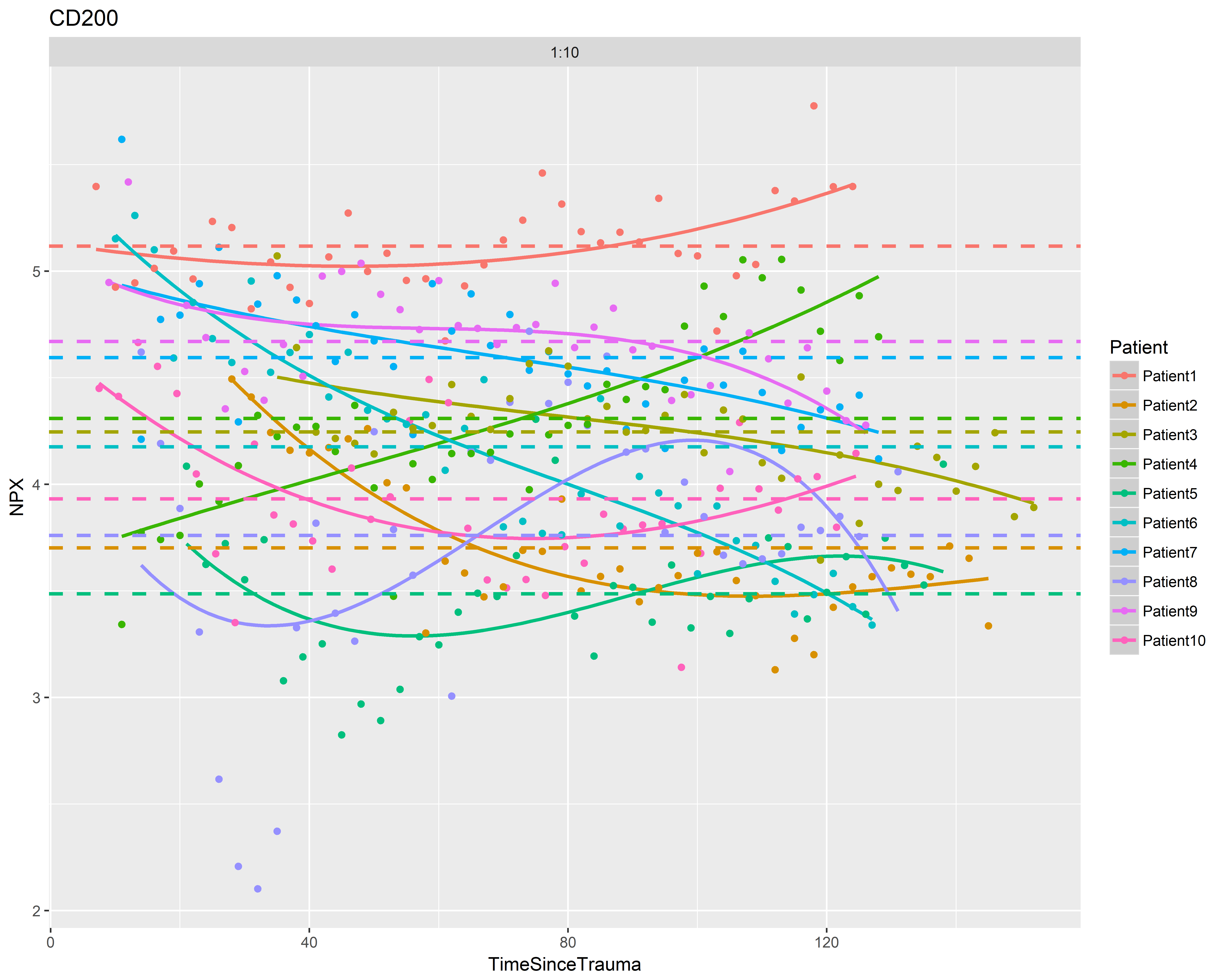


The figure demonstrates the temporal dynamics of CD200 the first 7 days post-injury in all 10 patients.

**Supplementary Figure 3. Temporal dynamics in CXCL10 the first 7 days post-injury – a line plot analysis**


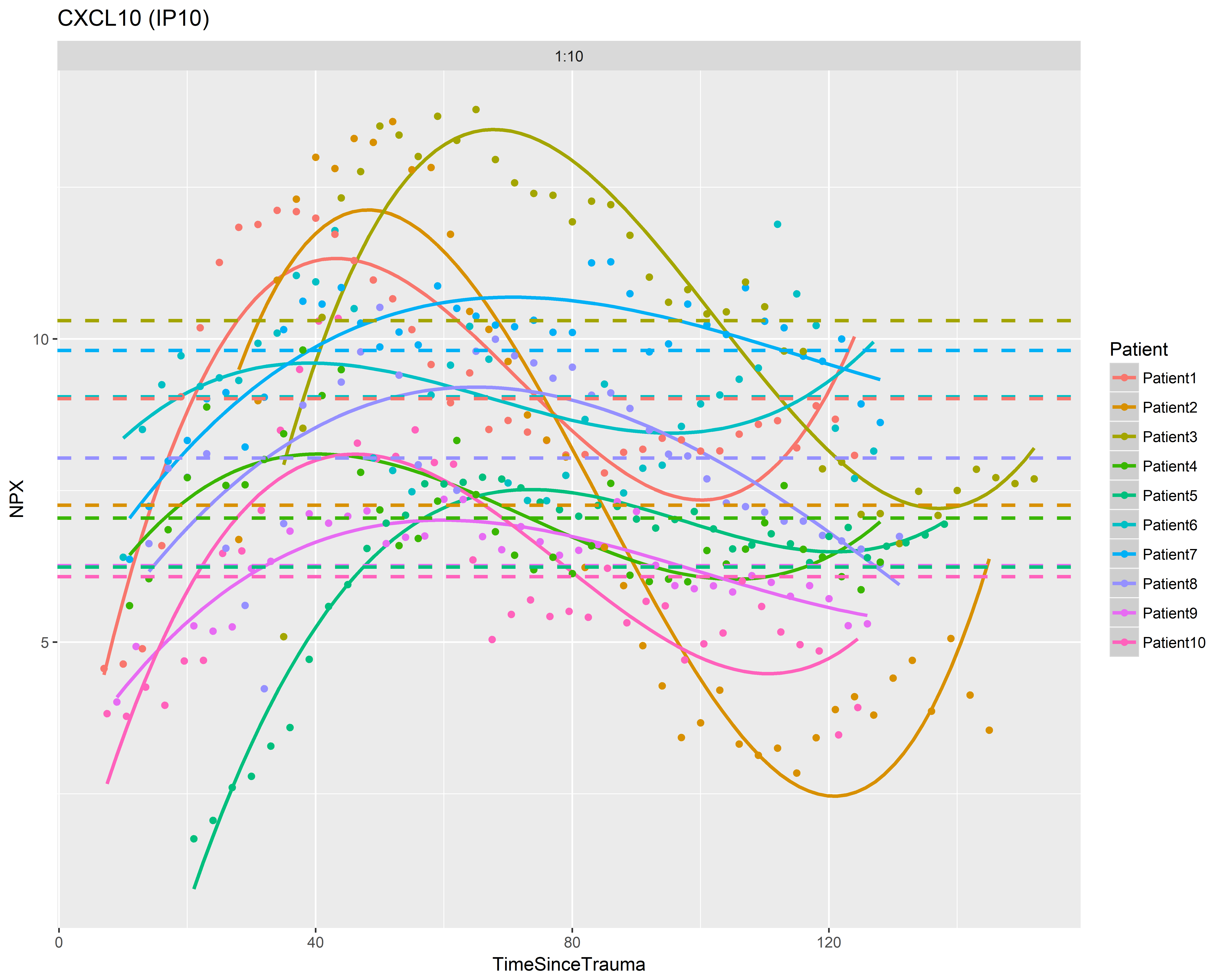


The figure demonstrates the temporal dynamics of CXCL10 the first 7 days post-injury in all 10 patients.

**Supplementary Figure 4. Temporal dynamics in DKK1 the first 7 days post-injury – a line plot analysis**


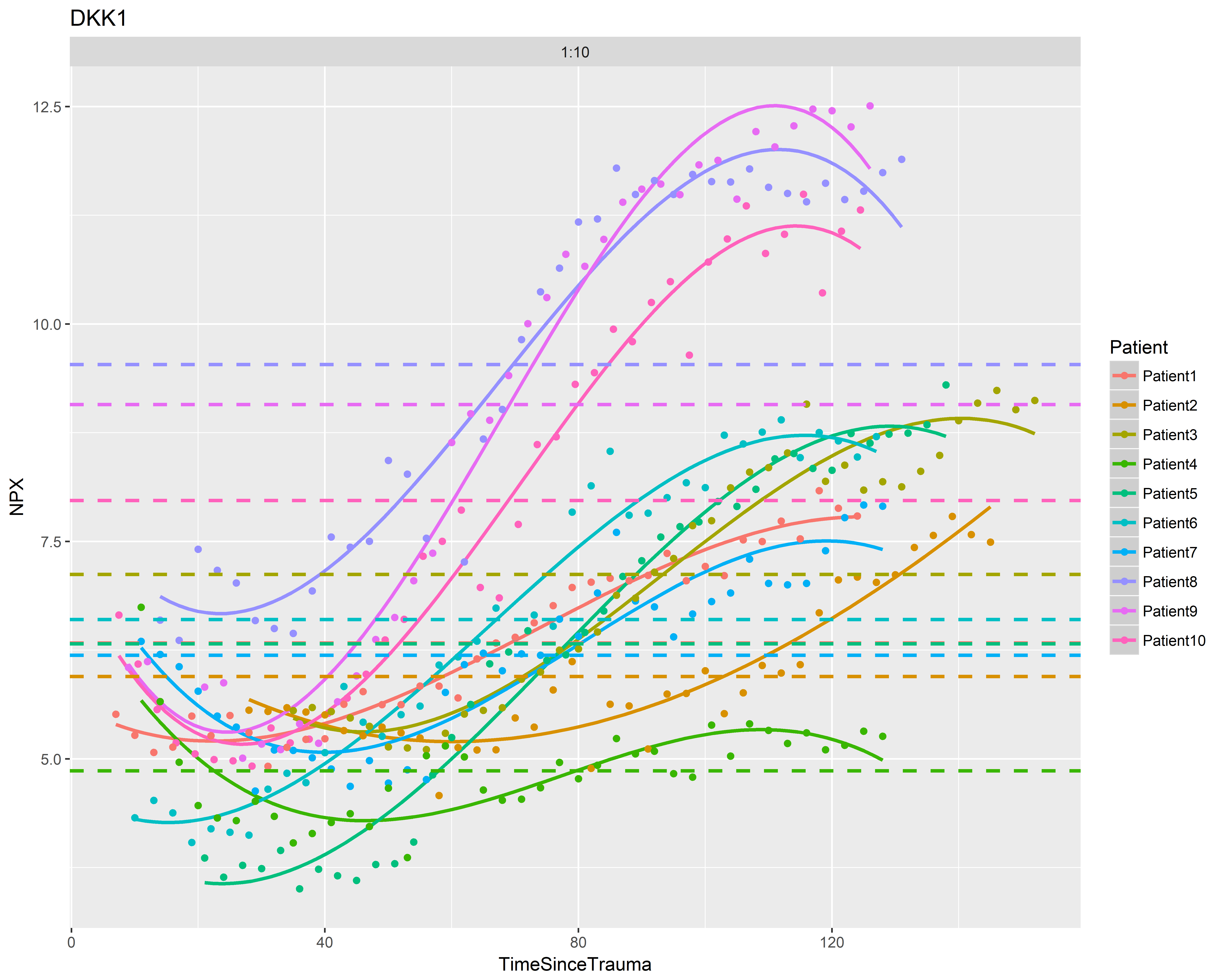


The figure demonstrates the temporal dynamics of DKK1 the first 7 days post-injury in all 10 patients.

**Supplementary Figure 5. Temporal dynamics in Draxin the first 7 days post-injury – a line plot analysis**


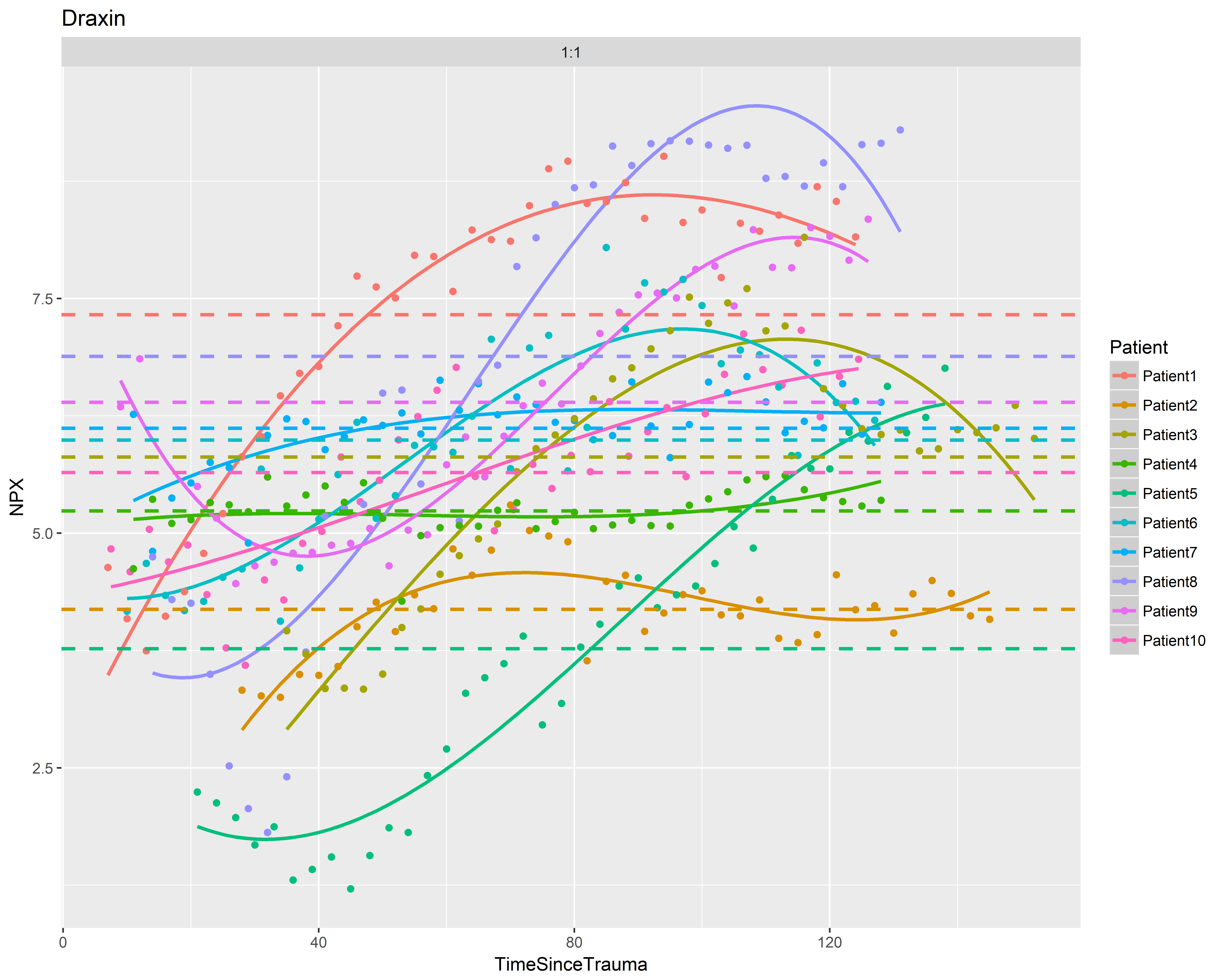


The figure demonstrates the temporal dynamics of Draxin the first 7 days post-injury in all 10 patients.

**Supplementary Figure 6. Temporal dynamics in G-CSF the first 7 days post-injury – a line plot analysis**


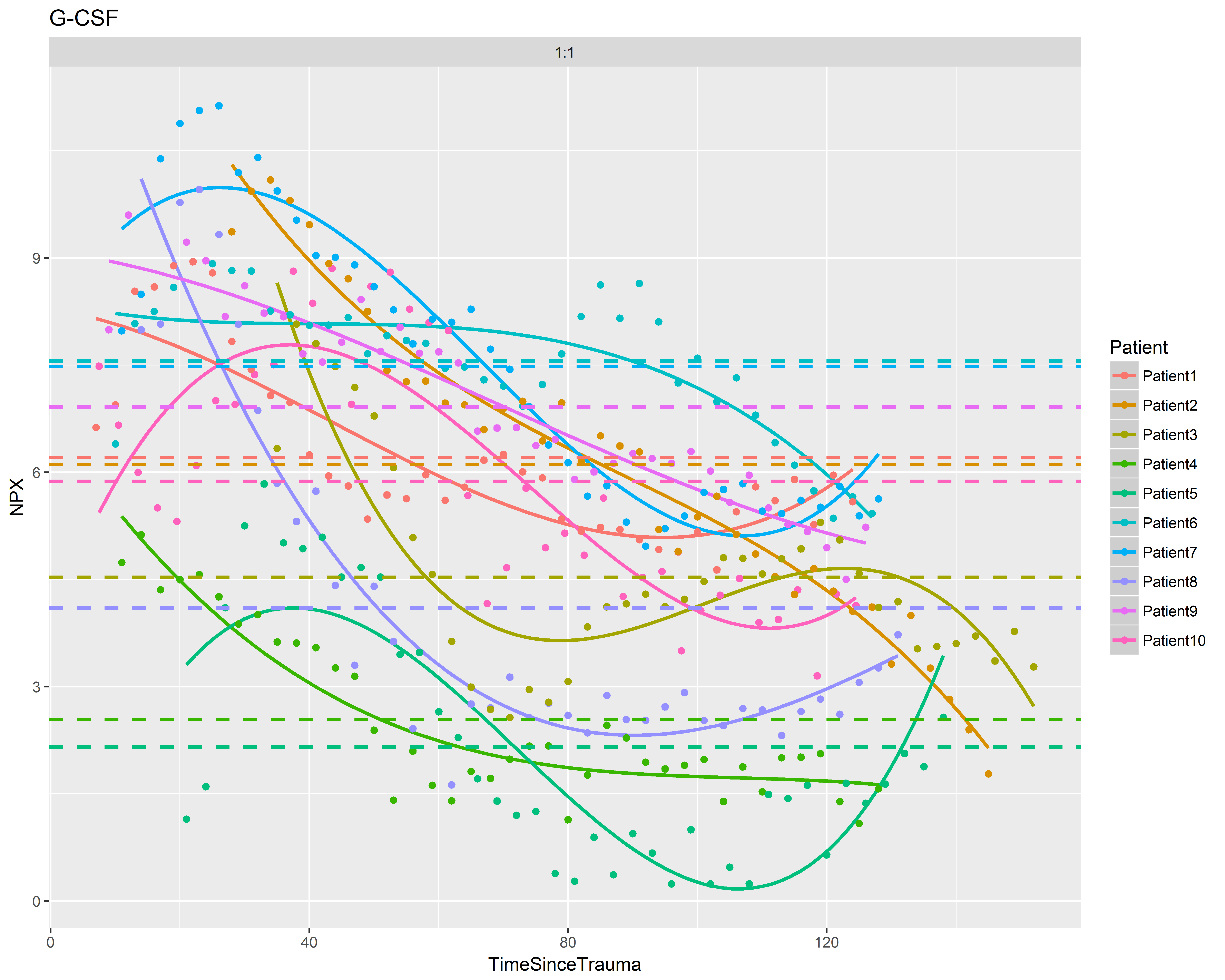


The figure demonstrates the temporal dynamics of G-CSF the first 7 days post-injury in all 10 patients.

**Supplementary Figure 7. Temporal dynamics in IL-1β the first 7 days post-injury – a line plot analysis**


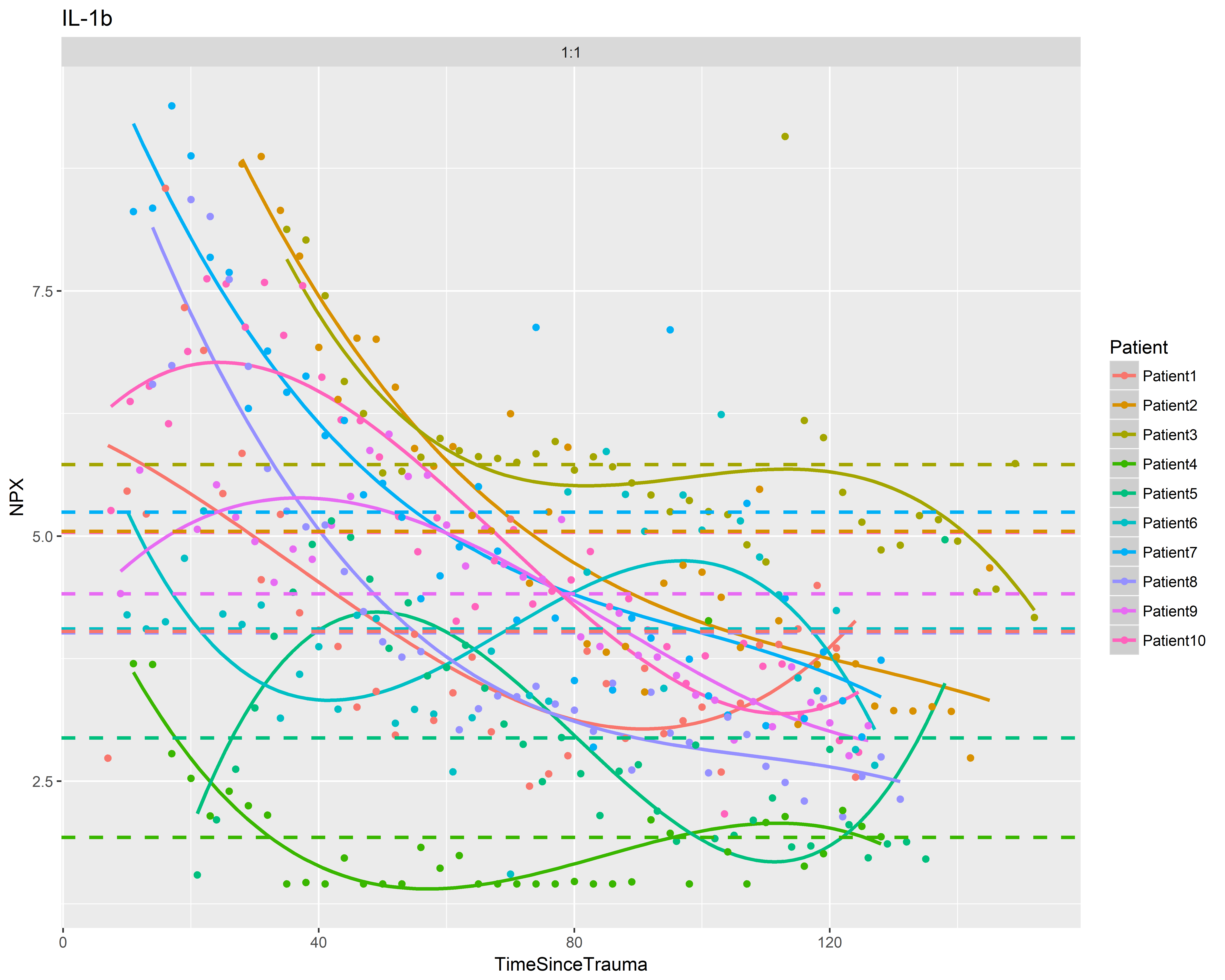


The figure demonstrates the temporal dynamics of IL-1β the first 7 days post-injury in all 10 patients.

**Supplementary Figure 8. Temporal dynamics in IL-1ra the first 7 days post-injury – a line plot analysis**


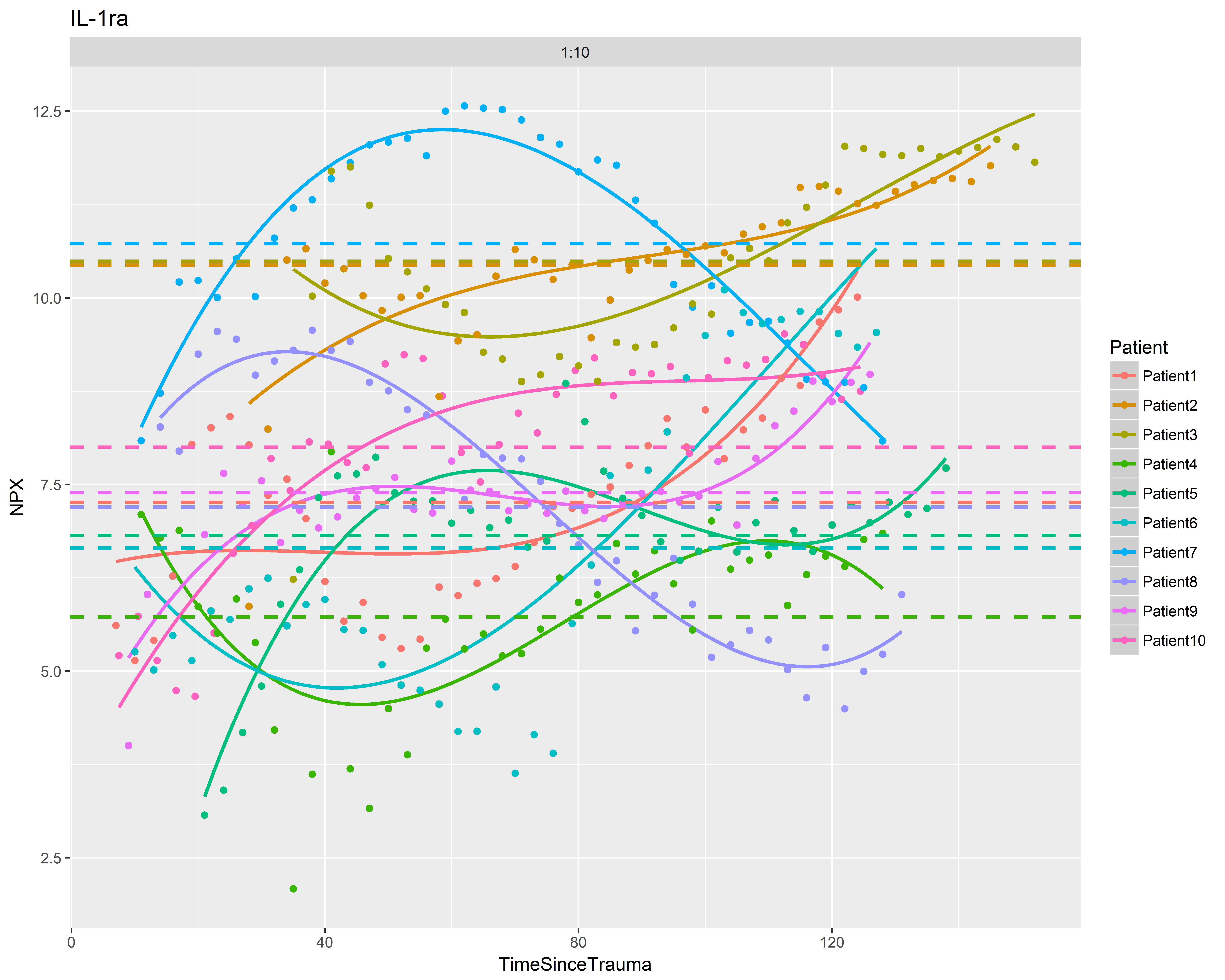


The figure demonstrates the temporal dynamics of IL-1ra the first 7 days post-injury in all 10 patients.

**Supplementary Figure 9. Temporal dynamics in IL-6 the first 7 days post-injury – a line plot analysis**


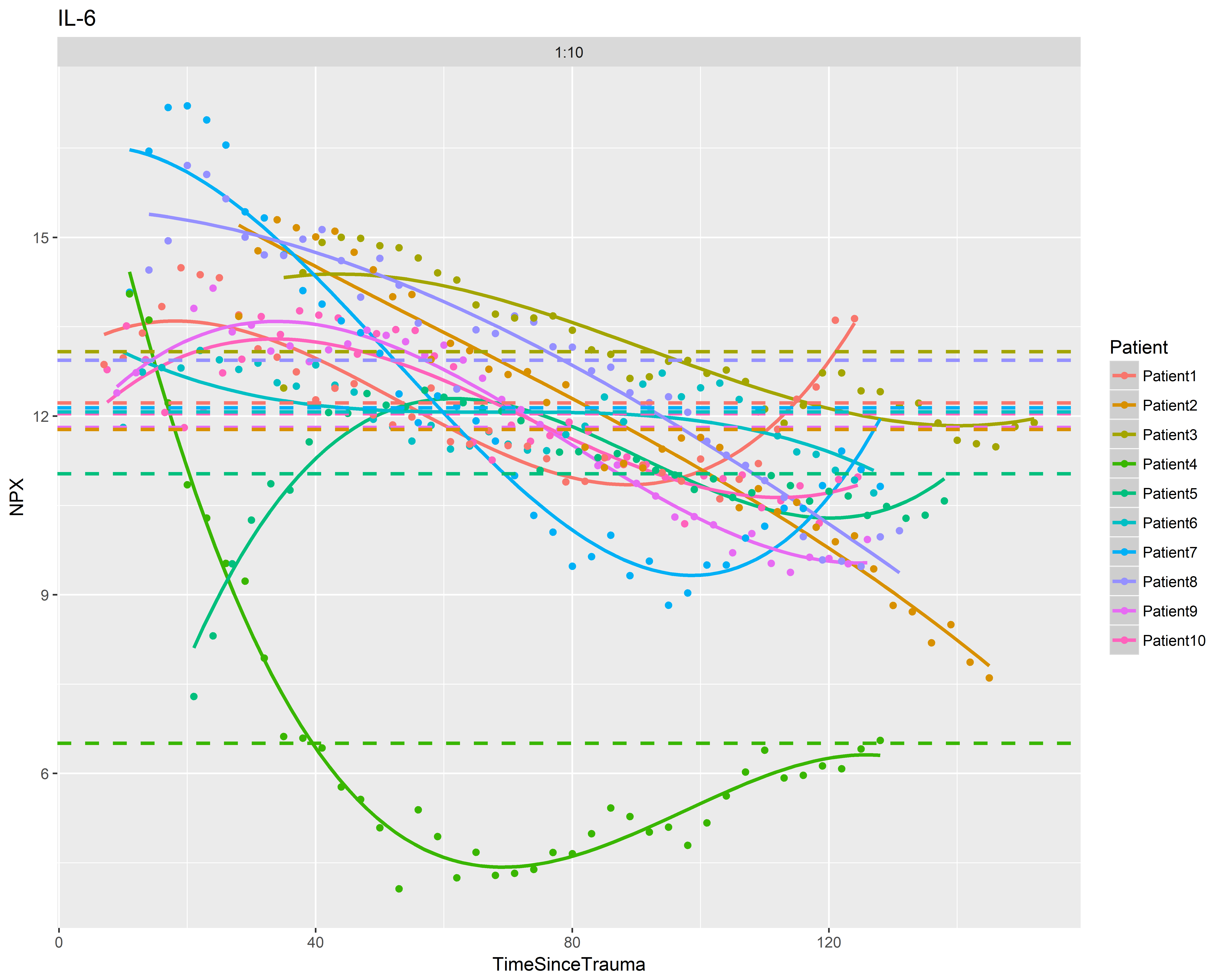


The figure demonstrates the temporal dynamics of IL-6 the first 7 days post-injury in all 10 patients.

**Supplementary Figure 10. Temporal dynamics in IL-8 the first 7 days post-injury – a line plot analysis**


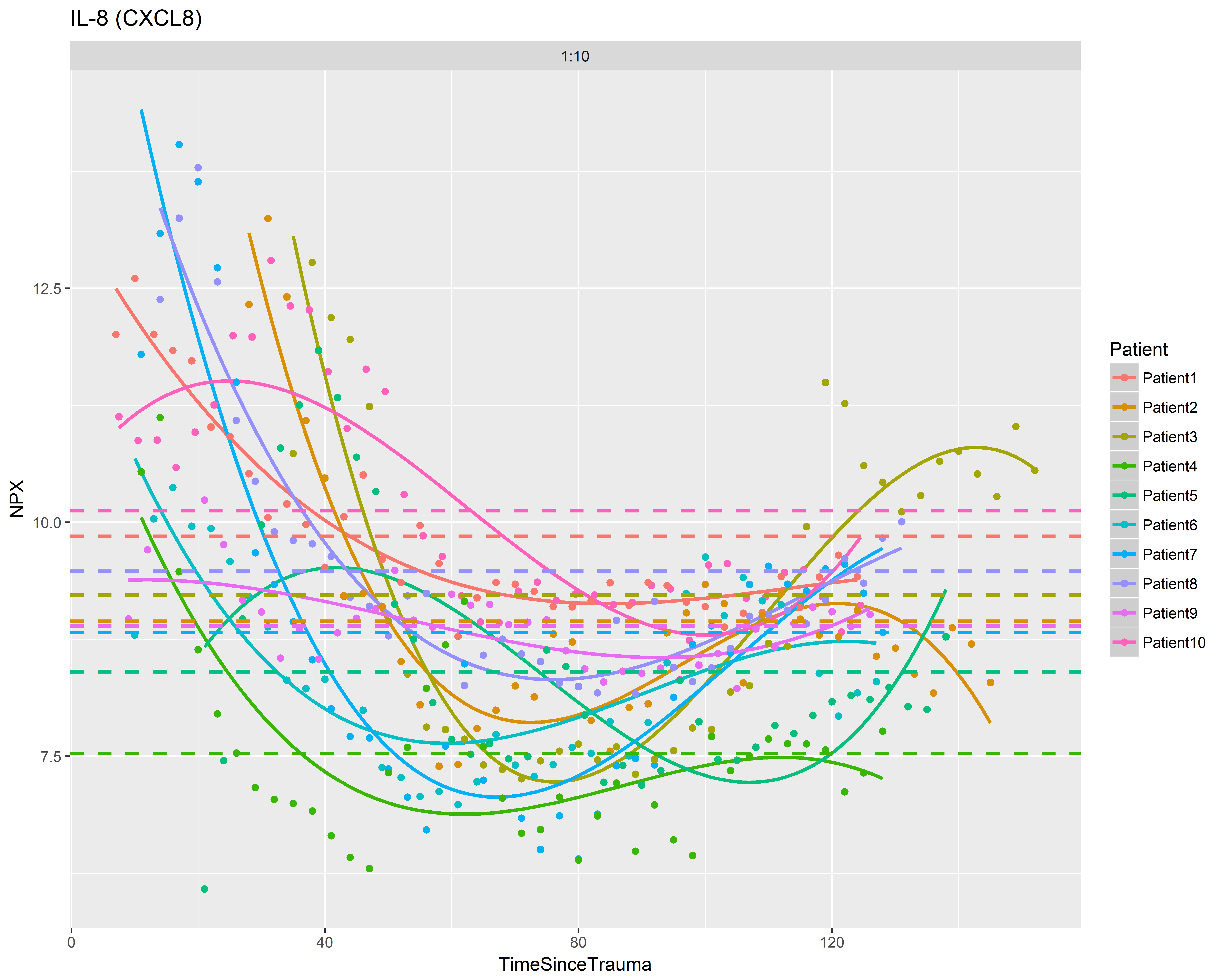


The figure demonstrates the temporal dynamics of IL-8 the first 7 days post-injury in all 10 patients.

**Supplementary Figure 11. Temporal dynamics in JAMB the first 7 days post-injury – a line plot analysis**


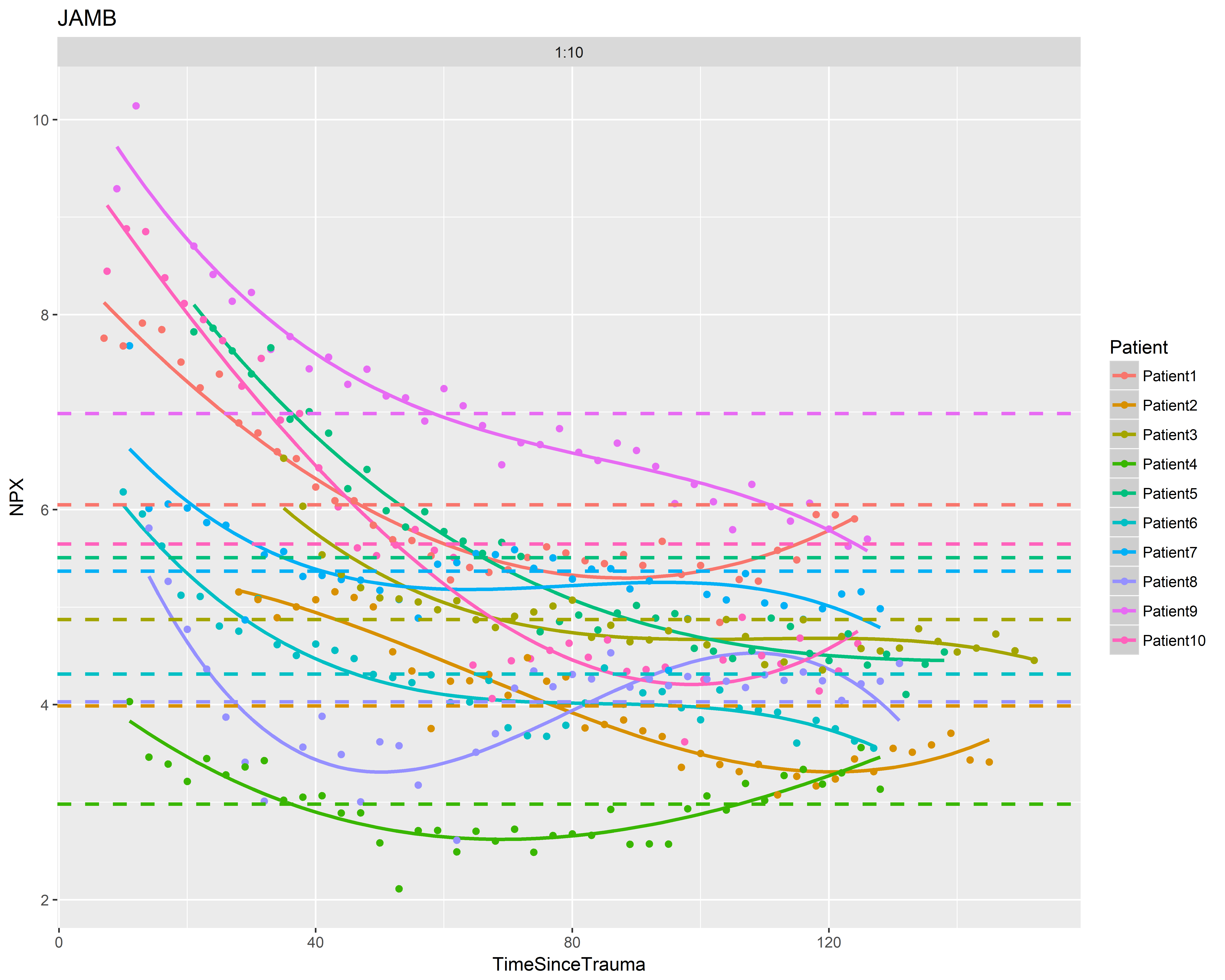


The figure demonstrates the temporal dynamics of JAMB the first 7 days post-injury in all 10 patients.

**Supplementary Figure 12. Temporal dynamics in MCP-2 the first 7 days post-injury – a line plot analysis**


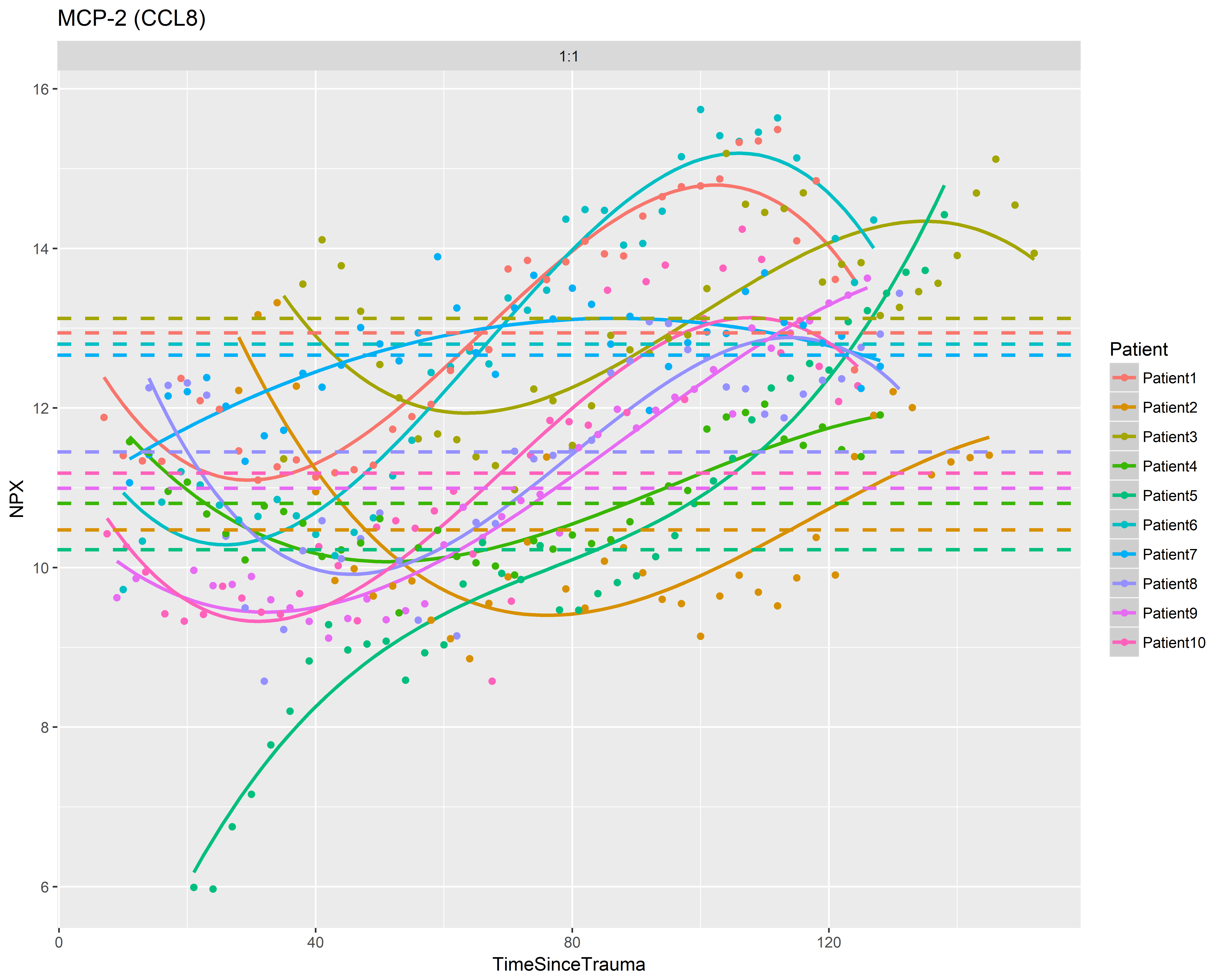


The figure demonstrates the temporal dynamics of MCP-2 the first 7 days post-injury in all 10 patients.

**Supplementary Figure 13. Temporal dynamics in MCP-3 the first 7 days post-injury – a line plot analysis**


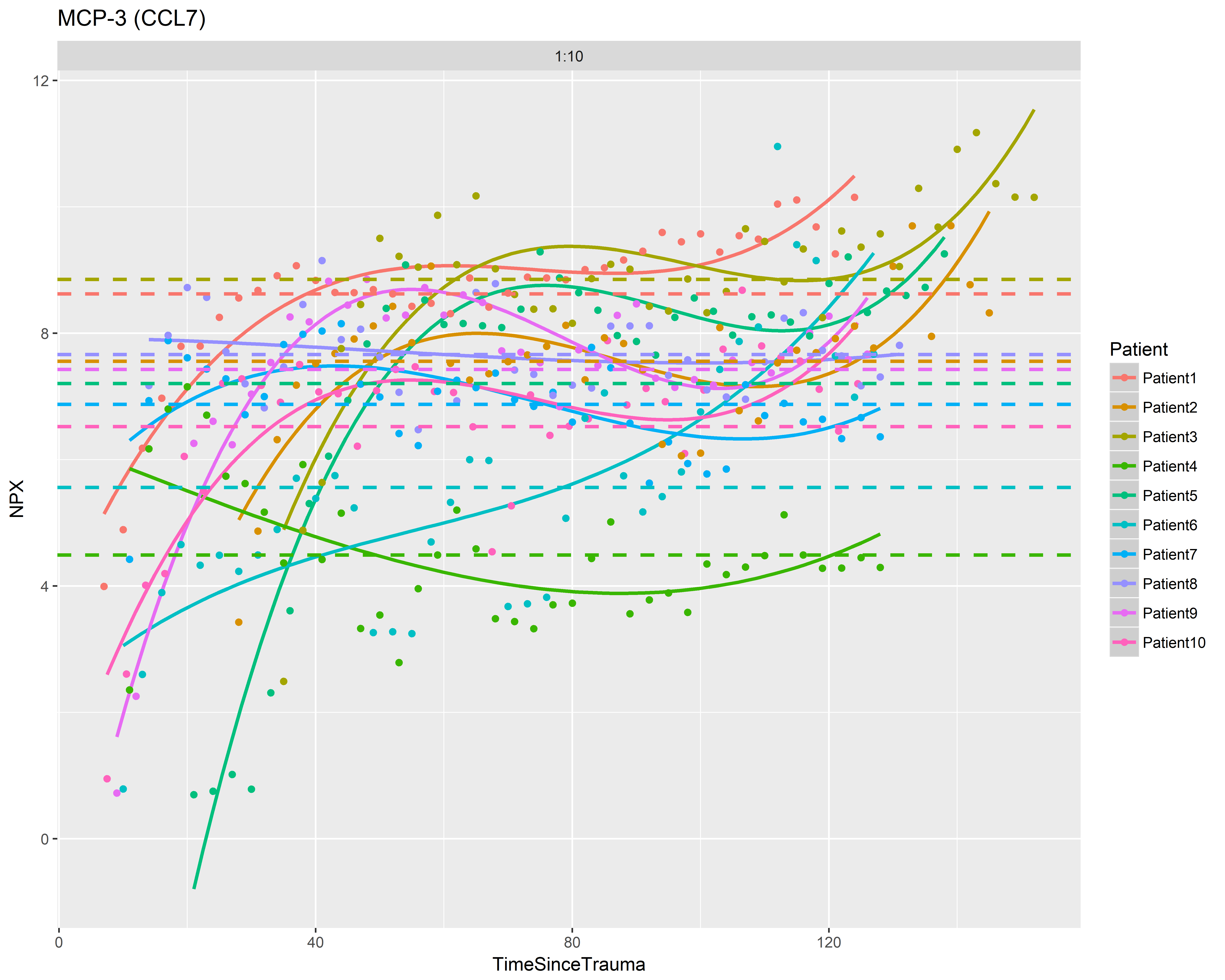


The figure demonstrates the temporal dynamics of MCP-3 the first 7 days post-injury in all 10 patients.

**Supplementary Figure 14. Temporal dynamics in MIP-1b the first 7 days post-injury – a line plot analysis**


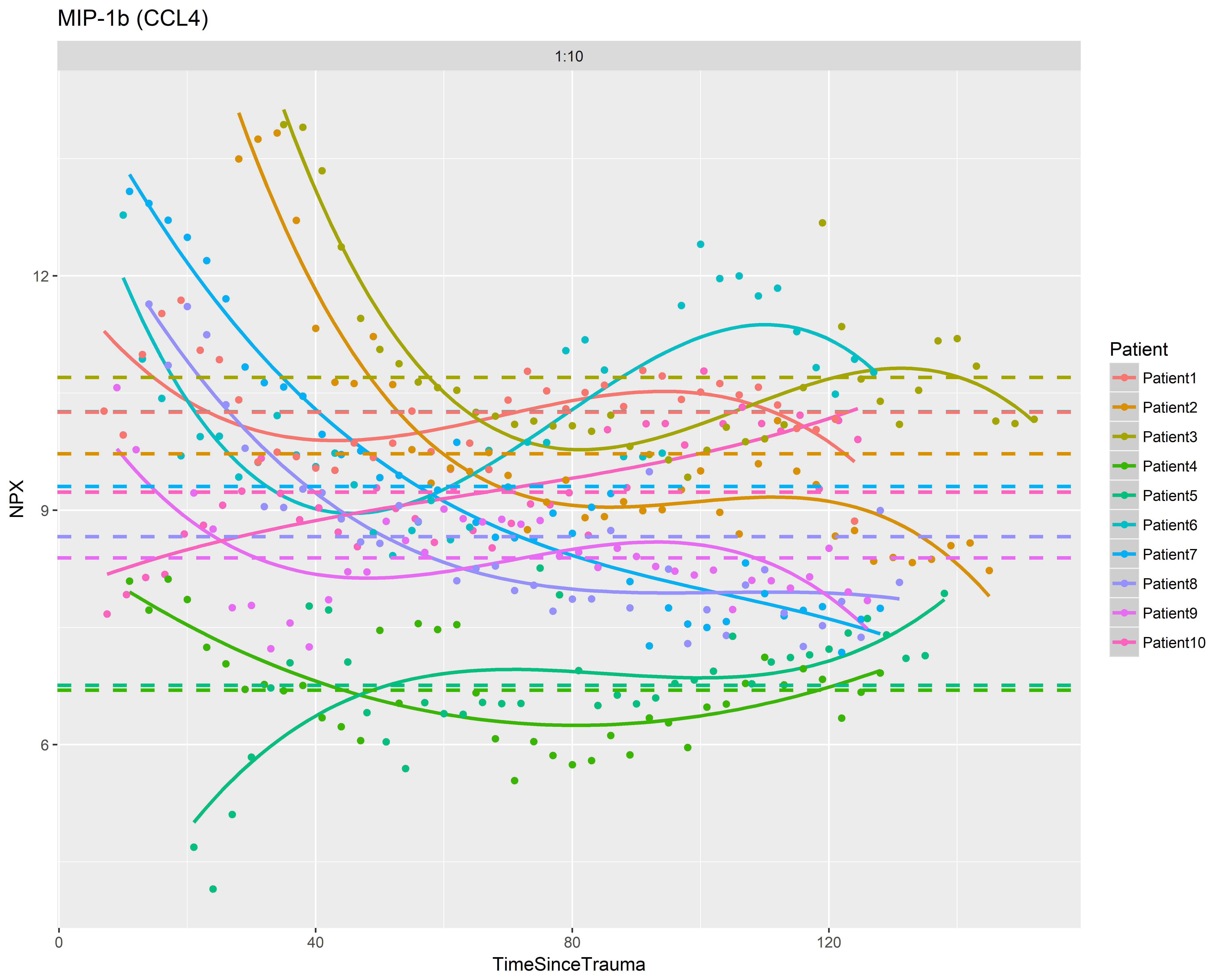


The figure demonstrates the temporal dynamics of MIP-1b the first 7 days post-injury in all 10 patients.

**Supplementary Figure 15. Temporal dynamics in NCAN the first 7 days post-injury – a line plot analysis**


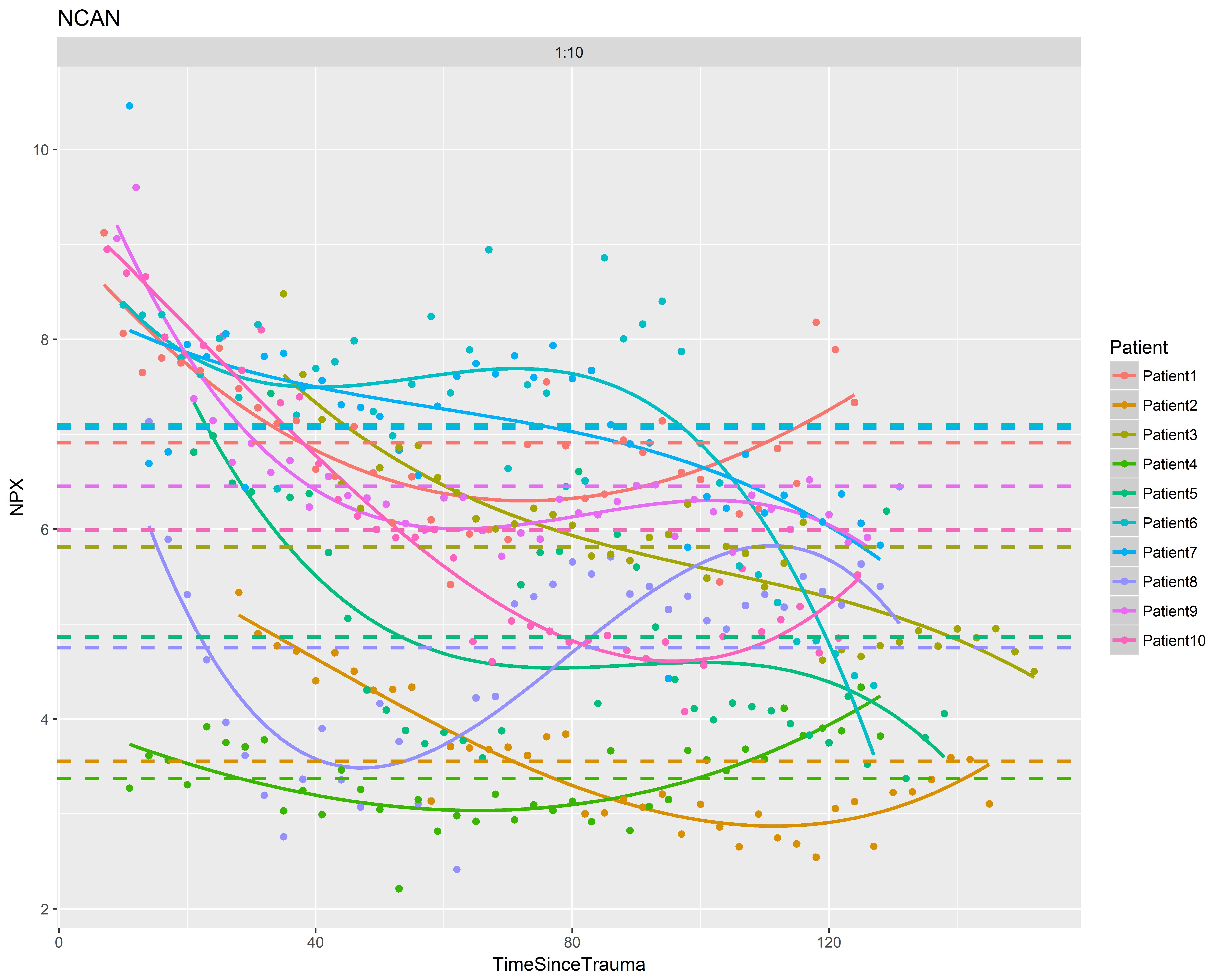


The figure demonstrates the temporal dynamics of NCAN the first 7 days post-injury in all 10 patients.

**Supplementary Figure 16. Temporal dynamics in NFH the first 7 days post-injury – a line plot analysis**


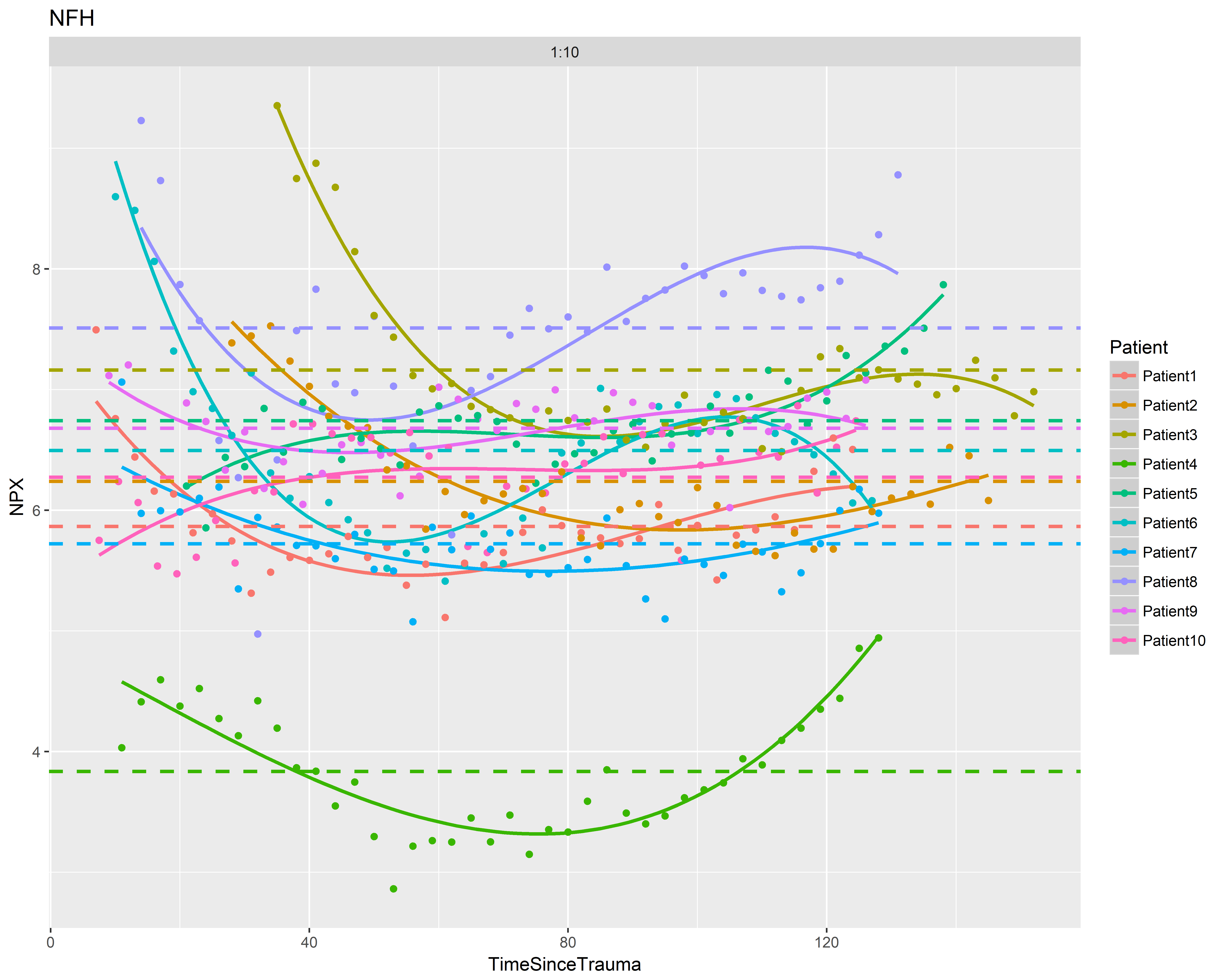


The figure demonstrates the temporal dynamics of NFH the first 7 days post-injury in all 10 patients.

**Supplementary Figure 17. Temporal dynamics in RGMA the first 7 days post-injury – a line plot analysis**


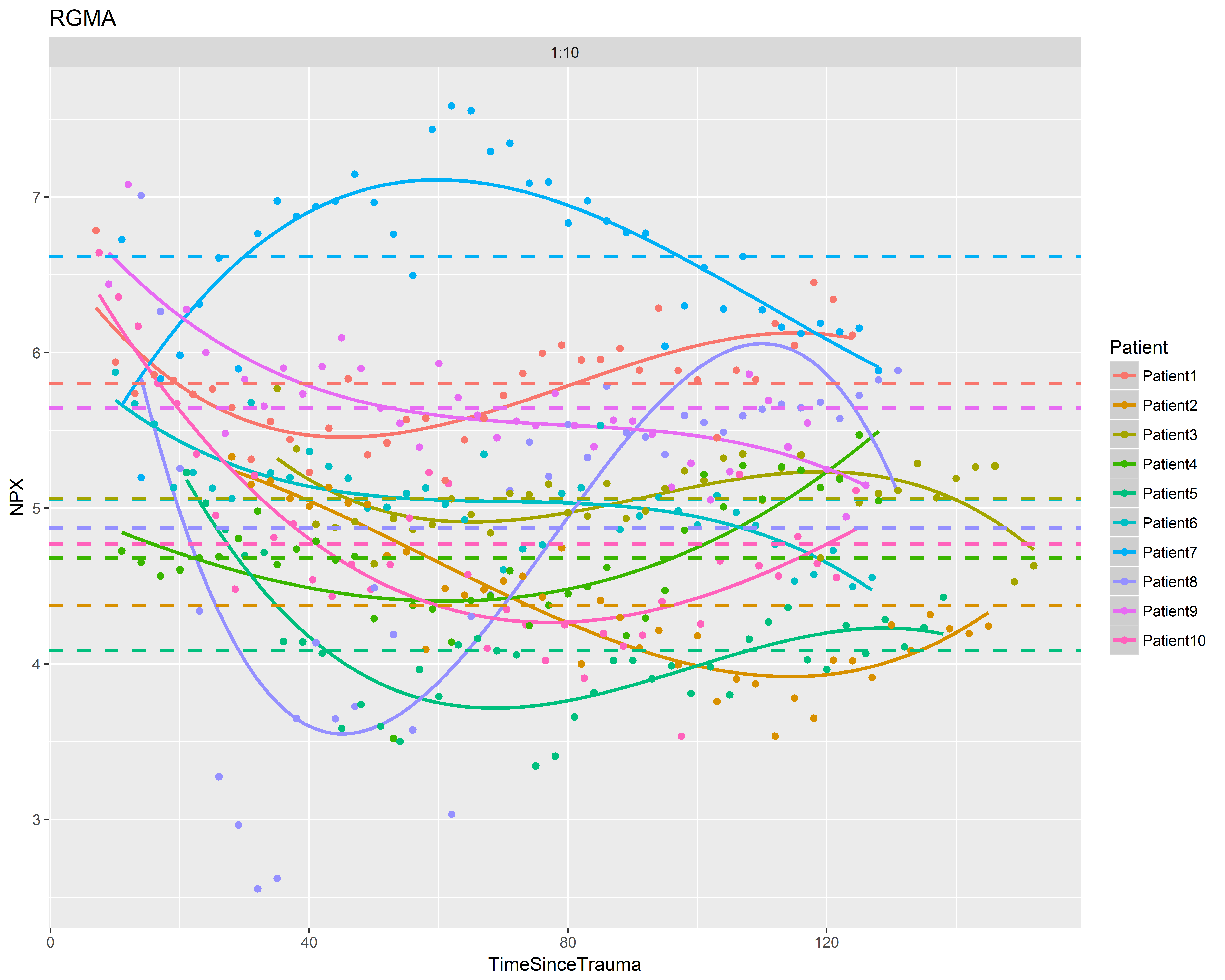


The figure demonstrates the temporal dynamics of RGMA the first 7 days post-injury in all 10 patients.

**Supplementary Figure 18. Temporal dynamics in TGF-α the first 7 days post-injury – a line plot analysis**


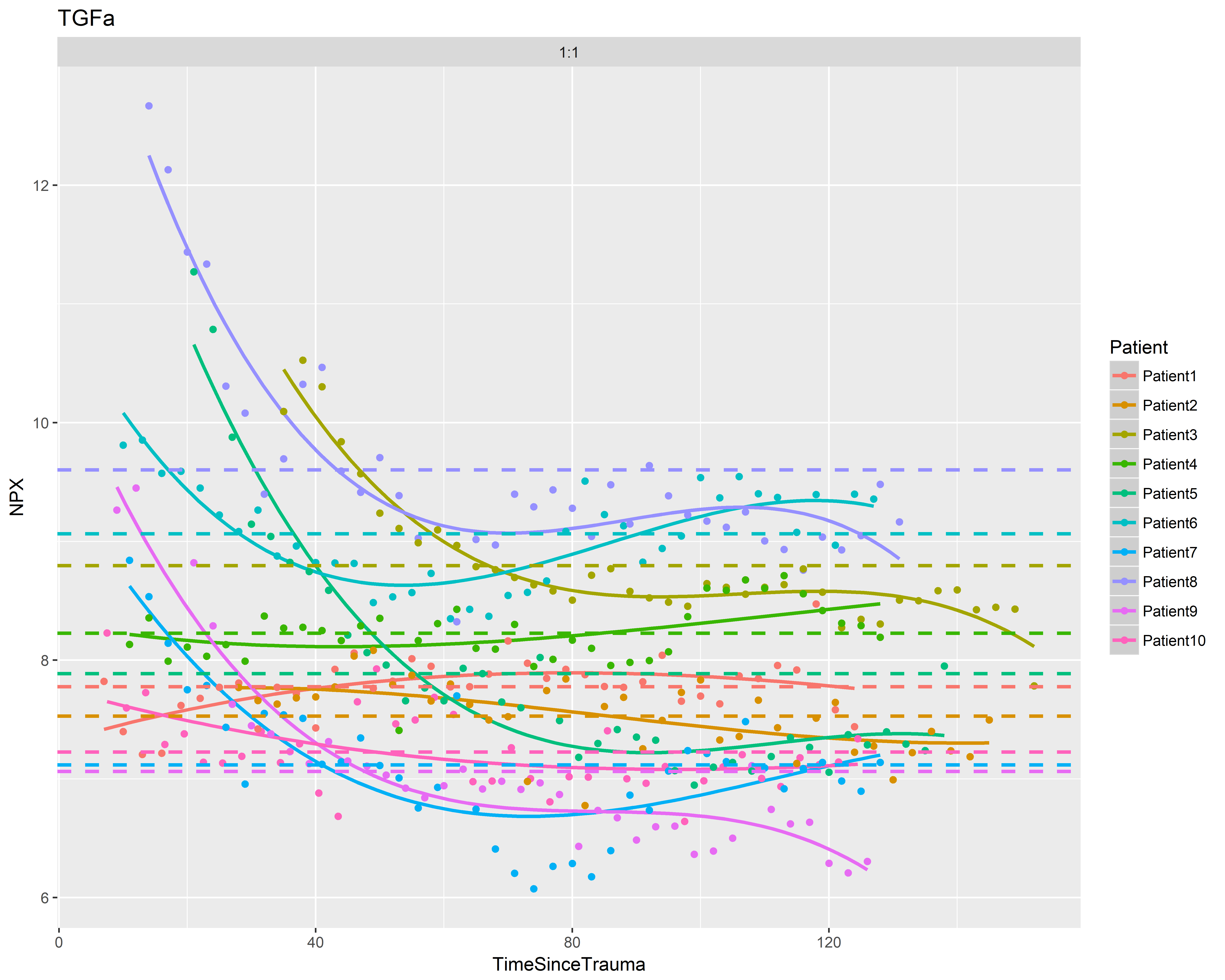


The figure demonstrates the temporal dynamics of TGF-α the first 7 days post-injury in all 10 patients.

**Supplementary Figure 19. Temporal dynamics in uPA the first 7 days post-injury – a line plot analysis**


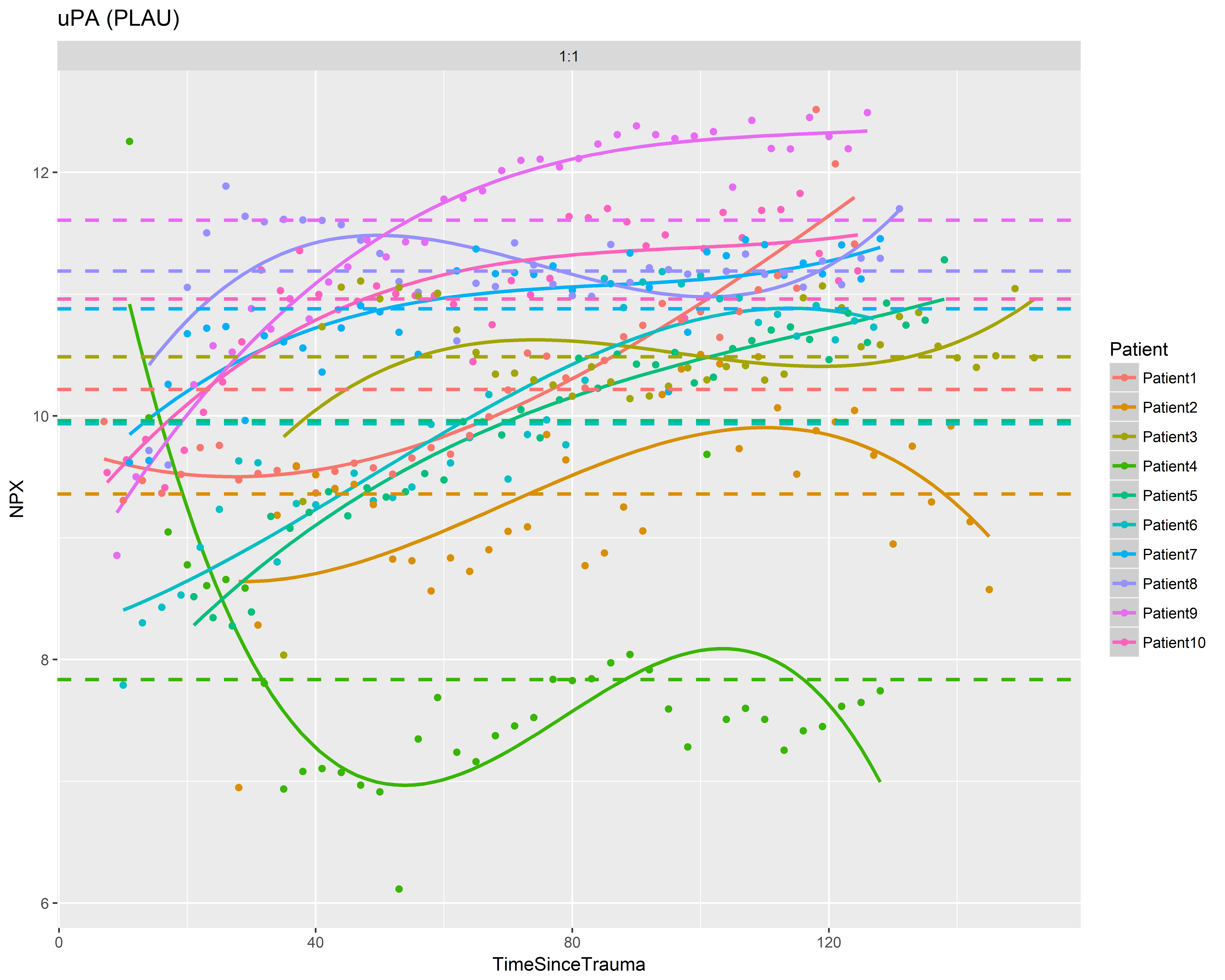


The figure demonstrates the temporal dynamics of uPA the first 7 days post-injury in all 10 patients.

**Supplementary Figure 20. Temporal dynamics in VEGF the first 7 days post-injury – a line plot analysis**


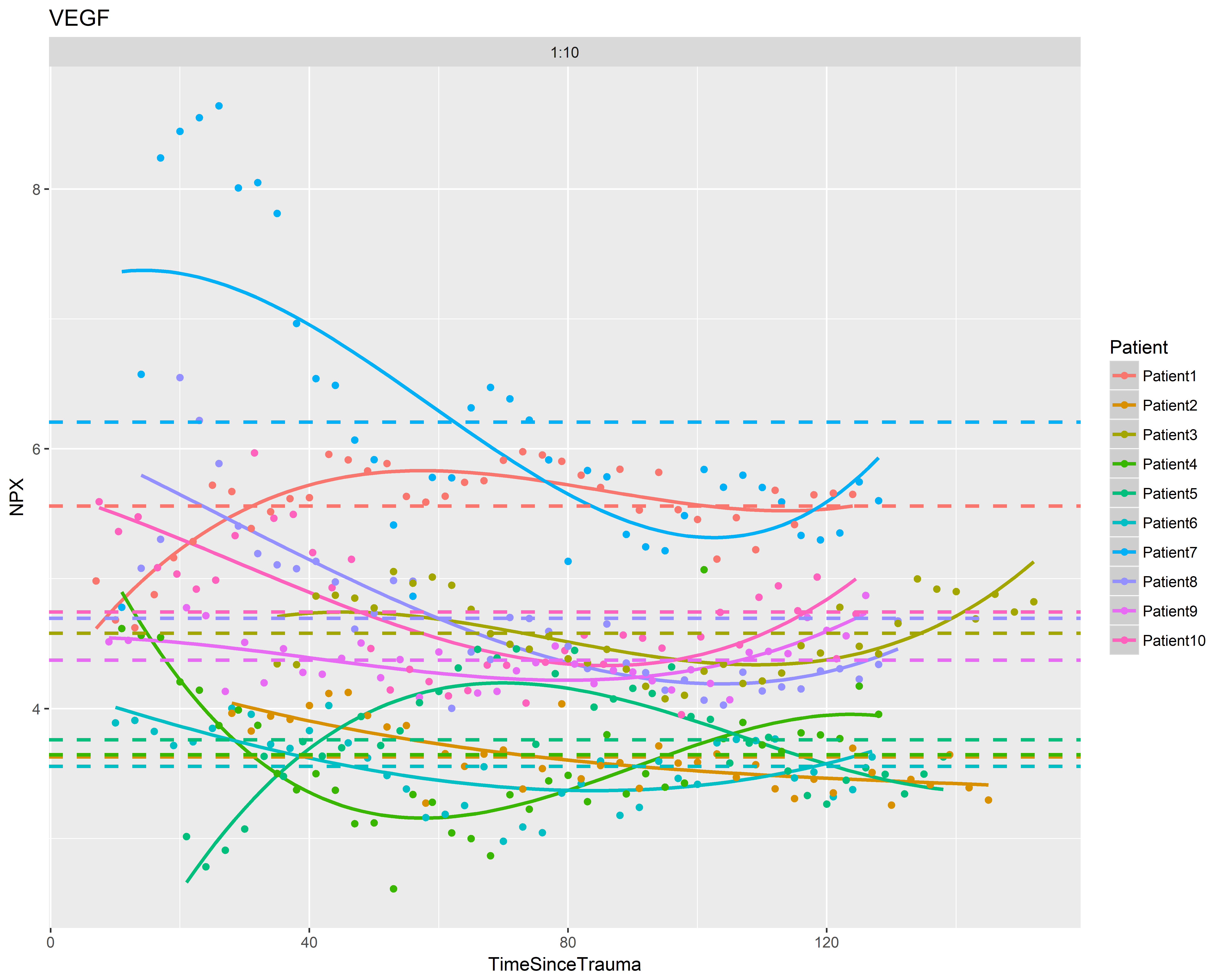


The figure demonstrates the temporal dynamics of VEFG the first 7 days post-injury in all 10 patients.
