# Supplementary material for: A Dedicated 21-Plex Proximity Extension Assay Panel for High-Sensitivity Protein Biomarker Detection Using Microdialysis in Severe Traumatic Brain Injury: The Next Step in Precision Medicine?
Source: Neurotrauma Rep. 2023 Jan 11;4(1):25–40. doi: 10.1089/neur.2022.0067 (PMC9886191; doi:10.1089/neur.2022.0067)
Supplement: Supplemental data [file Suppl_TableS2.docx]

**Supplementary table 2. Local brain tissue characteristics based on routine low molecular weight CMD biomarker data**

| Case no. | 1 | 2 | 3 | 4 | 5 | 6 | 7 | 8 | 9 | 10 |
| --- | --- | --- | --- | --- | --- | --- | --- | --- | --- | --- |
| Glucose <1mmol/L | 0% | **10%** | 7% | **13%** | 0% | 0% | **34%** | 2% | 0% | 4% |
| LPR >30 | 0% | **18%** | 1% | 9% | 0% | 10% | **35%** | **49%** | **30%** | **21%** |
| Lactate >4mmol/L | **10%** | **42%** | **34%** | **97%** | 2% | 0% | **99%** | **10%** | **100%** | 2% |
| Pyruvate <120μmol/L | 0% | **49%** | **47%** | **15%** | **55%** | **23%** | 0% | 5% | 3% | **94%** |
| Glutamate >15μmol/L | 2% | **10%** | 0% | **100%** | **44%** | 6% | **42%** | **100%** | **90%** | **14%** |
| Glycerol >100μmol/L | **16%** | **96%** | 1% | **32%** | 1% | **35%** | **21%** | **71%** | **63%** | **63%** |

The table shows the percentage of monitoring time with critical biomarker levels during the five days of CMD monitoring for the individual patients. Bold values indicate critical levels for 10% or more of the monitoring time.
